# NanoBondy Reaction through NeissLock Anhydride Allows Covalent Immune Cell Decoration

**DOI:** 10.1021/acs.bioconjchem.5c00519

**Published:** 2026-01-24

**Authors:** Lasya R. Vankayala, Kish R. Adoni, Sheryl Y. T. Lim, Tommy Dam, Omer Dushek, Konstantinos Thalassinos, Mark R. Howarth

**Affiliations:** † Department of Biochemistry, University of Oxford, South Parks Road, Oxford OX1 3QU, U.K.; ‡ Department of Pharmacology, 2152 University of Cambridge, Tennis Court Road, Cambridge CB2 1PD, U.K.; § Engineering Biology Interdisciplinary Research Centre, University of Cambridge, Cambridge CB2 1GA, U.K.; ∥ Sir William Dunn School of Pathology, 6396 University of Oxford, South Parks Road, Oxford OX1 3RE, U.K.; ⊥ Institute of Structural and Molecular Biology, Division of Biosciences, University College London, London WC1E 6BT, U.K.; # Institute of Structural and Molecular Biology, Birkbeck College, University of London, London WC1E 6BT, U.K.

## Abstract

Cell-surface conjugation has enormous therapeutic and research potential. Existing technologies for cell-surface modification are usually reversible, nonspecific, or rely on genetic editing of target cells. Here, we present the NanoBondy, a nanobody modified for covalent ligation to an unmodified protein target at the cell surface. The NanoBondy utilizes the 20 naturally occurring amino acids, harnessing NeissLock chemistry engineered from *Neisseria meningitidis*. We evaluated the binding and specificity of a panel of nanobodies to CD45, a long-lived surface marker of nucleated hematopoietic cells. We demonstrated the conversion of existing nanobodies to covalently reacting NanoBondies using a disulfide clamp to position the self-processing module of FrpA close to the nanobody antigen-binding site. The addition of calcium induces anhydride formation at the NanoBondy C-terminus, enabling proximity-directed ligation to surface amines on CD45. We optimized the NanoBondy reaction by fine-tuning linkers and disulfide clamp sites to modulate anhydride positioning. Tandem mass spectrometry mapped reaction sites between NanoBondy and CD45. NanoBondy ligation was robust to buffer, pH, and temperature and was detected within 2 minutes. We established the reaction specificity of NanoBondies to endogenous CD45 at the surface of NK cells and T cells. NanoBondy technology provides a modular approach for targeted, inducible, and covalent cell-surface modification of immune cells without their genetic modification.

## Graphical abstract

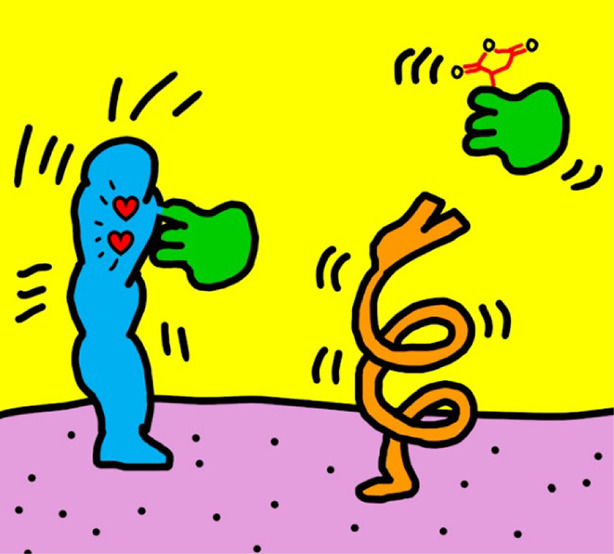

## Introduction

Molecular recognition in living systems is dominated by networks of noncovalent contacts. However, many applications in research and biotechnology are limited by the instability of such binding interactions.^1,2^ Instability may pose a challenge in response to harsh conditions or force, but it is most commonly an issue for long-lasting labeling, such as when attempting to change cell behavior *in vivo*.^3,4^ There has been particular excitement about cell-surface conjugation to enhance cell therapy, given the great success of chimeric antigen receptor (CAR)-T cells against leukemia and lymphoma.^5–7^ However, CAR-T cells have not yet fulfilled their potential in destroying solid tumors.^5–7^ To enhance therapeutic activity, T cells have been armed with cytokines, small molecule drugs, checkpoint inhibitors, or extracellular matrix-degrading enzymes, either directly^8–10^ or within nanoparticles^11–15^ or nanogels.^16^

Modular tags for covalent ligation (e.g., HaloTag,^17^ SNAP-tag,^18^ SpyTag/SpyCatcher,^19^ split intein,^20^ sortase^21^) have been valuable for cell-surface decoration.^22–24^ However, in cell therapy, the bacterial origin of most tag systems may raise immunogenicity concerns.^24,25^ Genetic modification of cell therapies also faces challenges, including the time required from transduction to surface expression, the potential for insertional mutagenesis, and innate immune activation caused by nucleic acid delivery.^26^ Each genetic change to cells adds to the delay, complexity, and cost of this exceptionally expensive therapeutic class.^27^ Cells may also be modified by inserting hydrophobic moieties into the plasma membrane, which is widely applicable but lacks specificity of insertion site or cell type.^28,29^ In addition, the hydrophobic tails gradually deinsert from the plasma membrane and can reinsert into neighboring cells.^30^ Covalent ligation has been achieved through metabolic labeling using amino acids or carbohydrates with bio-orthogonal groups, which leads to surface display for click reactions.^13,31–33^ Chemical cross-linkers^11,14,15^ or N-hydroxysuccinimide-based biotinylation followed by streptavidin labeling also allow stable cell decoration.^34^ However, such approaches modify multiple proteins, which may interfere with cell function.^4,35^

Nanobodies, also known as Variable Heavy domain of Heavy chain (VHH) or single-domain antibodies (sdAb), are a binding scaffold typically derived from immunizing llamas, alpacas, or camels. Nanobodies are an excellent platform for molecular engineering, given their small size, stability, high affinity, and ease of production in *Escherichia coli*.^36,37^ Unnatural amino acid mutagenesis has been used to generate covalently reactive nanobodies for the Sulfur Fluoride Exchange (SuFEx) reaction^31,32^ or the singlet oxygen-induced reaction of a furan warhead,^38^ but it faces challenges in scalability toward large-scale protein production.^39^ To create a new modality for covalent recognition of unmodified proteins, here we describe the NanoBondy, a covalently reactive nanobody harnessing our group’s NeissLock chemistry.^40^ NeissLock is engineered from the self-processing module (SPM) of FrpA^41^ of *Neisseria meningitidis*. Addition of calcium activates SPM, resulting in rapid autoproteolysis at an aspartate-proline bond. This step leads to formation of a highly electrophilic anhydride,^40,42^ which then can undergo attack by water or by nucleophiles such as amines on nearby proteins (Figure 1A). While the NeissLock SPM module is bacterially derived, calcium addition results in the release of the SPM moiety. As a result, the final conjugated adduct contains only a single amino acid scar: the Asp derived from the N-terminus of SPM. NeissLock has previously been used to lock together preexisting protein–protein interactions that are naturally present in a specific organism, where there is a high-resolution structure of the complex in the Protein Data Bank (PDB).^40^ Here, we advance NeissLock technology beyond endogenously occurring protein–protein pairs to artificial complexes where there is no experimental structure. By engineering the fusion of this SPM to a preexisting nanobody, with precise linkers and a disulfide clamp, we enable covalent conjugation of a nanobody to its protein target in an inducible and targeted manner, after which the SPM moiety can diffuse away (Figure 1B). We optimize NanoBondy conjugation in the context of the cell-surface target CD45, which is a long-lived and broadly expressed immune marker.^43^ We determine key features of the NanoBondy design for anhydride positioning and establish its robustness to reaction conditions for coupling to the isolated glycosylated extracellular domain. We then validate the NanoBondy coupling speed and specificity on cell lines and primary human immune cells. Extending the NanoBondy system, we then construct a DuoBondy, capable of covalent attachment to CD45, while including a second binder moiety allowing noncovalent attachment to the cancer checkpoint inhibitor target PD-1.

**Figure 1:**
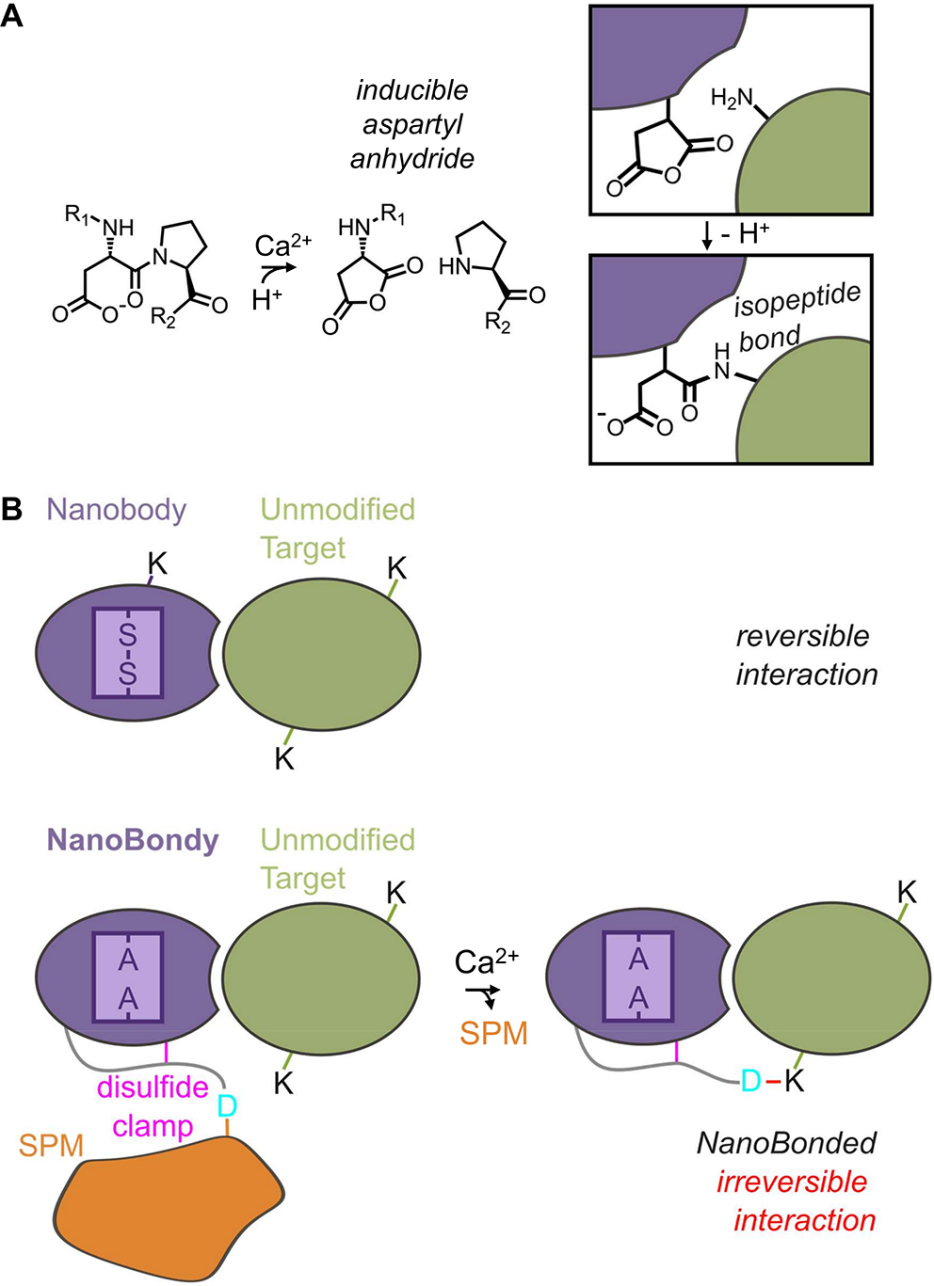
NanoBondy principle. (A) NeissLock chemistry. Upon the addition of calcium, the self-processing module (SPM) activates autoproteolysis at the aspartate–proline bond. This step generates a highly reactive aspartyl anhydride, which undergoes nucleophilic attack by a nearby nucleophilic amino acid or water. Fusing SPM to a binder (purple) thereby allows inducible covalent coupling to a target protein (green). (B) NanoBondy design. A nanobody (purple) employs complementarity-determining regions (CDRs) close to the N-terminus to bind its target (green). A regular nanobody can be engineered into a covalently reacting NanoBondy by inclusion of a flexible linker and disulfide clamp (magenta) to hold the reactive D (cyan) of SPM (orange) near the target, allowing anhydride-mediated covalent conjugation to the target after calcium activation.

## Results

### Selected Nanobody Candidates Demonstrate Specific Binding to CD45

Nanobodies to CD45 were previously generated from llama immunization.^44^ We selected 5 nanobodies that bind the d1d2 region of human CD45, furthest from the plasma membrane and conserved across isoforms of CD45.^44^ We cloned these nanobodies for bacterial expression with a C-terminal SpyTag003.^45^ All five nanobodies were solubly expressed in *E. coli* and were efficiently purified using SpySwitch affinity chromatography^46^ (Figure 2A).

**Figure 2:**
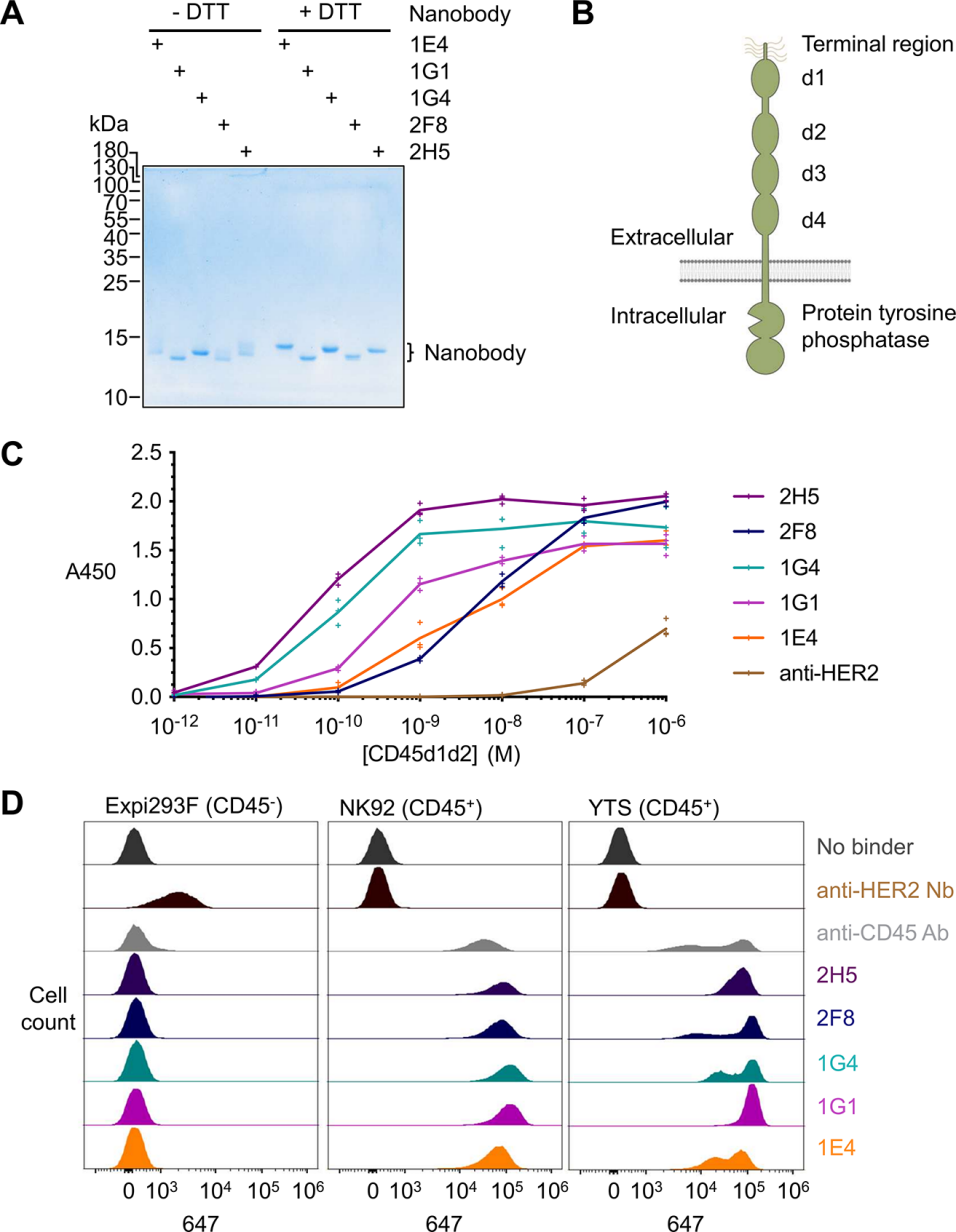
Characterization of anti-CD45 nanobodies. (A) Purification of anti-CD45 nanobodies. Nanobodies were expressed in *E. coli* and purified by SpySwitch affinity chromatography, followed by SDS-PAGE ± dithiothreitol (DTT) and Coomassie staining to assess disulfide bond formation. The experiment was conducted once. (B) Schematic of the organization of CD45, including extracellular domains d1–d4. (C) Binding of nanobodies to purified CD45. Nanobodies were coated on a plate and incubated with the indicated concentration of biotinylated human CD45 domains 1 and 2 (CD45d1d2), followed by colorimetric ELISA detection (absorbance at 450 nm). Anti-HER2 nanobody was used as a negative control. Each triplicate data point is shown with a line connecting the mean. Representative ELISA data were obtained from two independent experiments. (D) Binding of anti-CD45 nanobodies at the cell surface by flow cytometry. Anti-CD45 nanobodies were incubated with Expi293F, NK92, or YTS cells. Nanobody binding was detected using anti-VHH-Alexa Fluor 647. Anti-CD45 antibody was used as a positive control, with anti-HER2 nanobodies or unstained (no binder) cells as negative controls. Representative flow cytometry data were obtained from two independent experiments for Expi293F and YTS and one experiment with all three cell lines.

We expressed a recombinant fragment of the d1 and d2 domains of human CD45 (Figure 2B) linked to an AviTag for site-specific biotinylation (CD45d1d2) in Expi293F cells. Nanobody binding to CD45d1d2 was evaluated by an enzyme-linked immunosorbent assay (ELISA). All nanobodies demonstrated high-affinity binding to CD45d1d2, with 2H5 demonstrating the best affinity (Figure 2C). The negative control anti-HER2 nanobody showed negligible background binding to CD45d1d2 (Figure 2C).

Nanobody binding to endogenous human CD45 at the cell surface was tested by flow cytometry. Nanobodies were incubated with the YTS and NK92 natural killer (NK) cell lines (each CD45^+^), using Expi293F cells (CD45^–^) as a negative control. All nanobodies demonstrated binding to both CD45^+^ NK cell lines, with minimal nonspecific binding to CD45^–^ cells (Figure 2D). The anti-HER2 nanobody served as a positive control, and the HER2^+^ Expi293F cells could be stained successfully (Figure 2D). For further NanoBondy development, we prioritized 2H5 as the highest-affinity binder based on ELISA, as well as high-level and specific staining in flow cytometry.

### Designed NanoBondies Demonstrate Specific, Inducible Coupling to Purified CD45

In the absence of experimental structures for complexes with these nanobody binders, we utilized AlphaFold2-multimer^47,48^ and ParaFold^49^ to predict docking of a 2H5-derived NanoBondy to CD45d1d2 (Figure 3A). A NanoBondy is generated by fusing FrpA SPM to the nanobody’s C-terminus via a flexible linker containing a cysteine clamp to position the C-terminal anhydride of the NanoBondy close to the target for reaction (Figure 1B). Before the Asp-Pro cleavage site, we place a Gly-Ser-Tyr linker, which we previously established as optimal for rapid, high-yielding cleavage and anhydride generation.^40^

**Figure 3:**
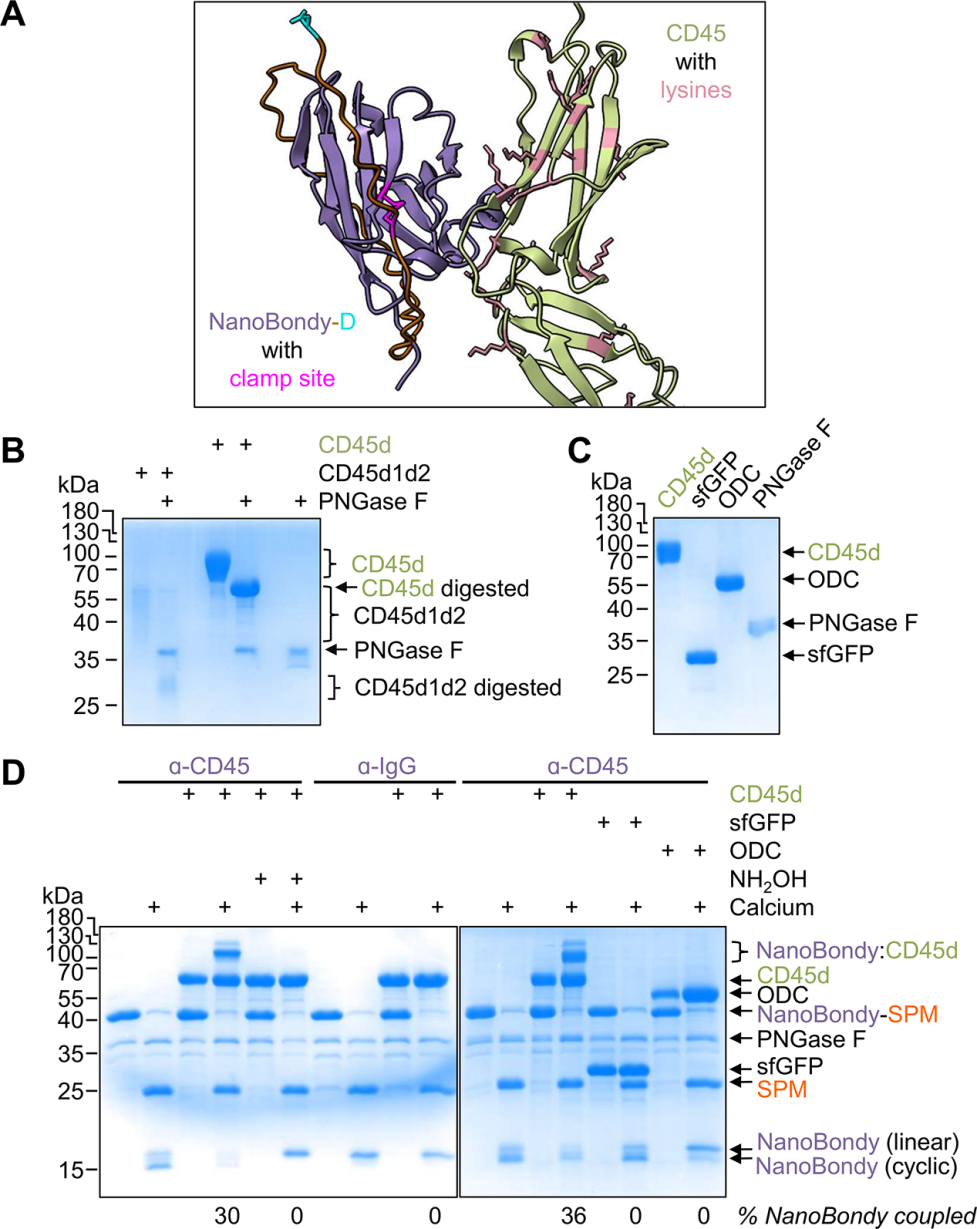
NanoBondy covalent conjugation to recombinant CD45. (A) AlphaFold prediction of 2H5 nanobody (purple) interaction with CD45d1d2 (green). Magenta represents the site for a disulfide clamp, with lysines on CD45d1d2 colored pink and the terminal aspartate in cyan. (B) MBP fusion improved the CD45 gel-based analysis. CD45d consists of MBP fused to domains 1 and 2 of CD45. PNGase F digestion decreased heterogeneous mobility of CD45d1d2 and CD45d upon SDS-PAGE with Coomassie staining. (C) Individual protein components for the conjugation assay in (D). CD45d, sfGFP, ODC, and PNGase F were validated by SDS-PAGE/Coomassie staining. (D) Specificity of the NanoBondy reaction with recombinant CD45. Anti-CD45 2H5 R72C or anti-IgG NanoBondy-SPM was incubated with CD45d, each at 10.5 μM, for 1 h at 37 °C in HBS ± calcium, followed by SDS-PAGE with Coomassie staining. ODC, sfGFP, and anti-IgG NanoBondy were used as negative controls for reaction specificity. Hydroxylamine was used as a competing nucleophile to block reactivity. Colon represents a covalent conjugate. The experiment was conducted once.

Based on the AlphaFold model, R72C on the nanobody was identified as the initial site for the disulfide clamp. Two endogenous cysteines in the nanobody, which form the core disulfide bond, were mutated to alanine to minimize any potential disulfide mispairing (Figure 1B). The designed NanoBondy (amino acid sequence in Figure S1) was expressed solubly in *E. coli* and purified using either C-tag or Ni-NTA purification. This NanoBondy was further validated by intact protein electrospray ionization mass spectrometry (Figure S2).

To allow simpler discrimination of reactant and product bands in gel assays, we cloned CD45d, which consists of CD45d1d2 with the maltose-binding protein (MBP) fused at its C-terminus. CD45d expresses well in Expi293F cells, yielding 88 mg per liter of culture (Figure 3B). Both CD45d1d2 and CD45d exhibit extensive N-linked glycosylation. We showed that Peptide:N-Glycosidase F (PNGase F) digestion facilitated analysis by SDS-PAGE (Figure 3B).

To test the 2H5 R72C NanoBondy reactivity to CD45d, the NanoBondy was mixed with CD45d or irrelevant protein targets in equimolar concentrations. We induced conjugation for 1 h at 37 °C with 2 mM CaCl_2_, comparable in concentration to the extracellular medium.^50^ The NanoBondy demonstrated calcium-inducible covalent conjugation to CD45d, which was competed out by the strong nucleophile hydroxylamine (NH_2_OH) (Figure 3D). We have previously shown that hydroxylamine quenches the SPM-derived anhydride.^40^ We generated a negative control NanoBondy by fusion of SPM to the anti-IgG nanobody TP1170. Covalent conjugation showed specificity for the anti-CD45 NanoBondy, with no product band observed when the anti-IgG NanoBondy was mixed with CD45d in the presence of calcium (Figure 3D). The anti-CD45 NanoBondy did not show conjugation to the noncognate protein targets superfolder GFP (sfGFP, expected NanoBondy covalent product mass of 44.5 kDa) or ornithine decarboxylase (ODC, expected NanoBondy covalent product mass of 70.6 kDa) (Figure 3C,D). We also showed the generality of converting different nanobodies to NanoBondies, demonstrating the specificity of coupling to CD45d by a NanoBondy based on a separate anti-CD45 nanobody, 2F8 (Figure S3).

### The NanoBondy Clamp Site and Linker Length Alter Conjugation Yield

We tested 3 alternative clamp sites in our NanoBondy design (Figure 4A), with clamp sites arranged in a tripod-like format, so that the resultant anhydride could sample various surfaces on the target. The reaction of NanoBondy with CD45d at different sites yielded different reaction bands that were resolved by SDS-PAGE. The abundance of each reaction band was altered by the choice of clamp site, with R72C demonstrating the highest reaction efficiency (Figure 4B), as evaluated by gel densitometry.

**Figure 4:**
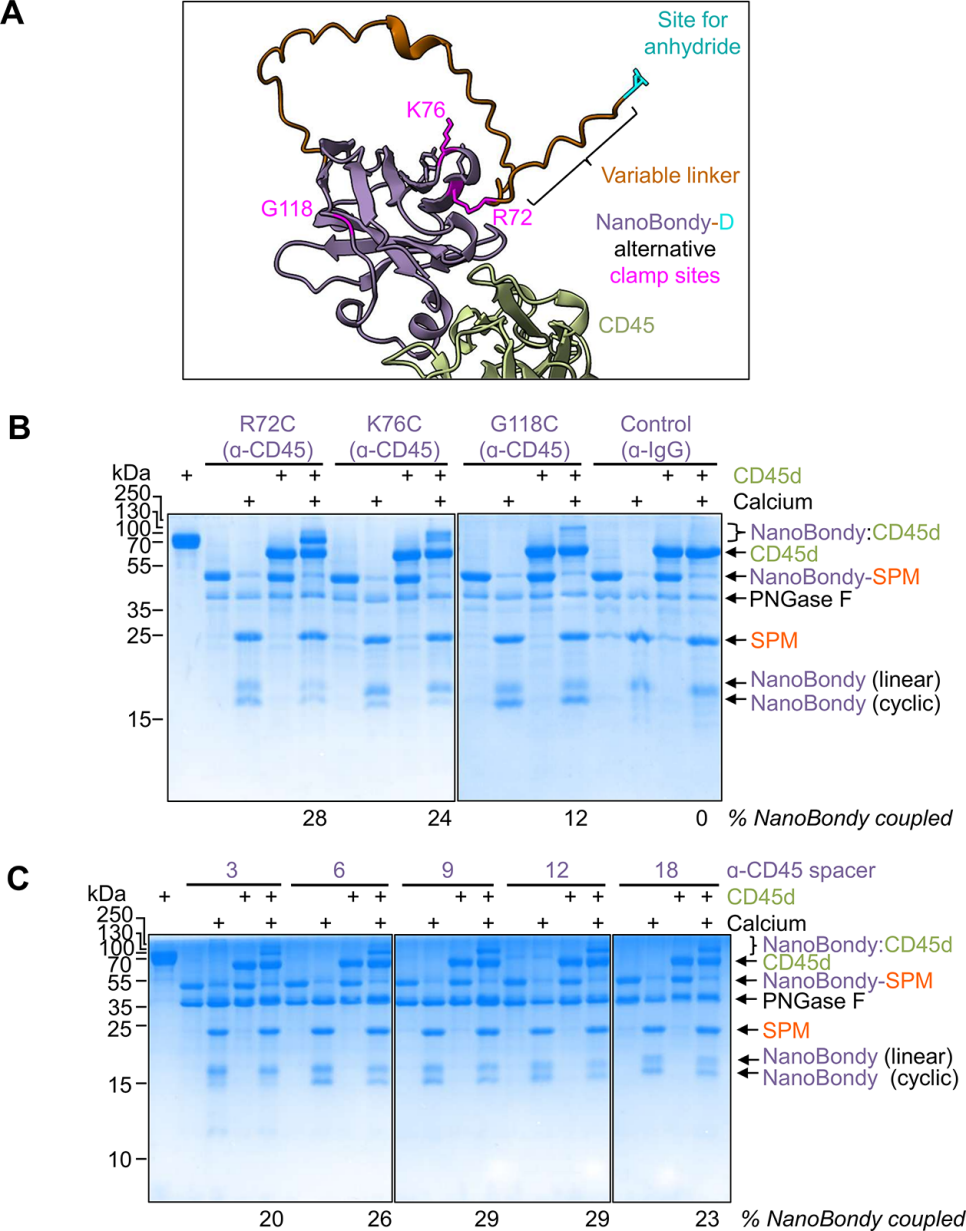
Optimization of NanoBondy clamp site and linker length. (A) AlphaFold prediction of 2H5 NanoBondy (purple) bound to CD45d1d2 (green). The reactive anhydride is shown in cyan, alternative clamp sites are shown in magenta, and linkers are shown in orange. (B) Clamp-site variant reactivity. 2H5 NanoBondy variants were incubated with CD45d at 10.5 μM each for 1 h at 37 °C in HBS ± Ca^2+^, followed by SDS-PAGE with Coomassie staining. Anti-IgG NanoBondy was used as a negative control. The leftmost lane represents CD45d without PNGase F treatment. The experiment was conducted once. (C) Linker variant reactivity for 2H5 R72C anti-CD45 NanoBondy, analyzed as in (B). Representative gels were obtained from two independent experiments.

We then varied the length of the flexible linker between the cysteine clamp site and the start of the SPM, testing lengths from 3 to 18 residues, to allow the anhydride to access more distant nucleophilic sites on the target. Interestingly, NanoBondy coupling to the CD45d target was still efficient despite these large changes in linker length (Figure 4C). By gel densitometry, we determined that the overall conjugation yield decreased when 3- and 6-residue linkers were employed. Increasing the linker length beyond 9 residues, however, did not improve conjugation efficiency. From these analyses, we selected the clamp site R72C and a 9-residue linker for further exploration.

### The NanoBondy Retains Reactivity across Various Conditions

Next, we evaluated the condition-dependence of the NanoBondy reaction, testing covalent conjugation to its target under various situations involving buffer, temperature, and pH. The NanoBondy was incubated with CD45d in the presence of calcium for varying durations before analysis by SDS-PAGE/Coomassie. The conjugation product was visible within 2 min under most conditions. The reaction proceeded more efficiently in HEPES Buffered Saline (HBS) than Tris-Buffered Saline (TBS) (Figure 5A). The reaction was faster at 37 °C than at 25 °C, with the majority of conjugation completed within 5 min at 37 °C (Figure 5A). We also evaluated pH-dependence by conducting the reaction in HBS along with 2-(N-morpholino)­ethanesulfonic acid (MES), which enables effective buffering over a wider pH range. NanoBondy reactivity was retained at pH 6.5–8.5, but the reaction proceeded more slowly at pH 8.5 than at pH 6.5 or 7.5 (Figure 5B). From these analyses, the optimal buffer conditions for the NanoBondy reaction are HBS at 37 °C and pH 7.5. Phosphate Buffered Saline (PBS) is not advised for NanoBondy reactions: the addition of calcium to activate the reaction would result in calcium phosphate precipitation.

**Figure 5:**
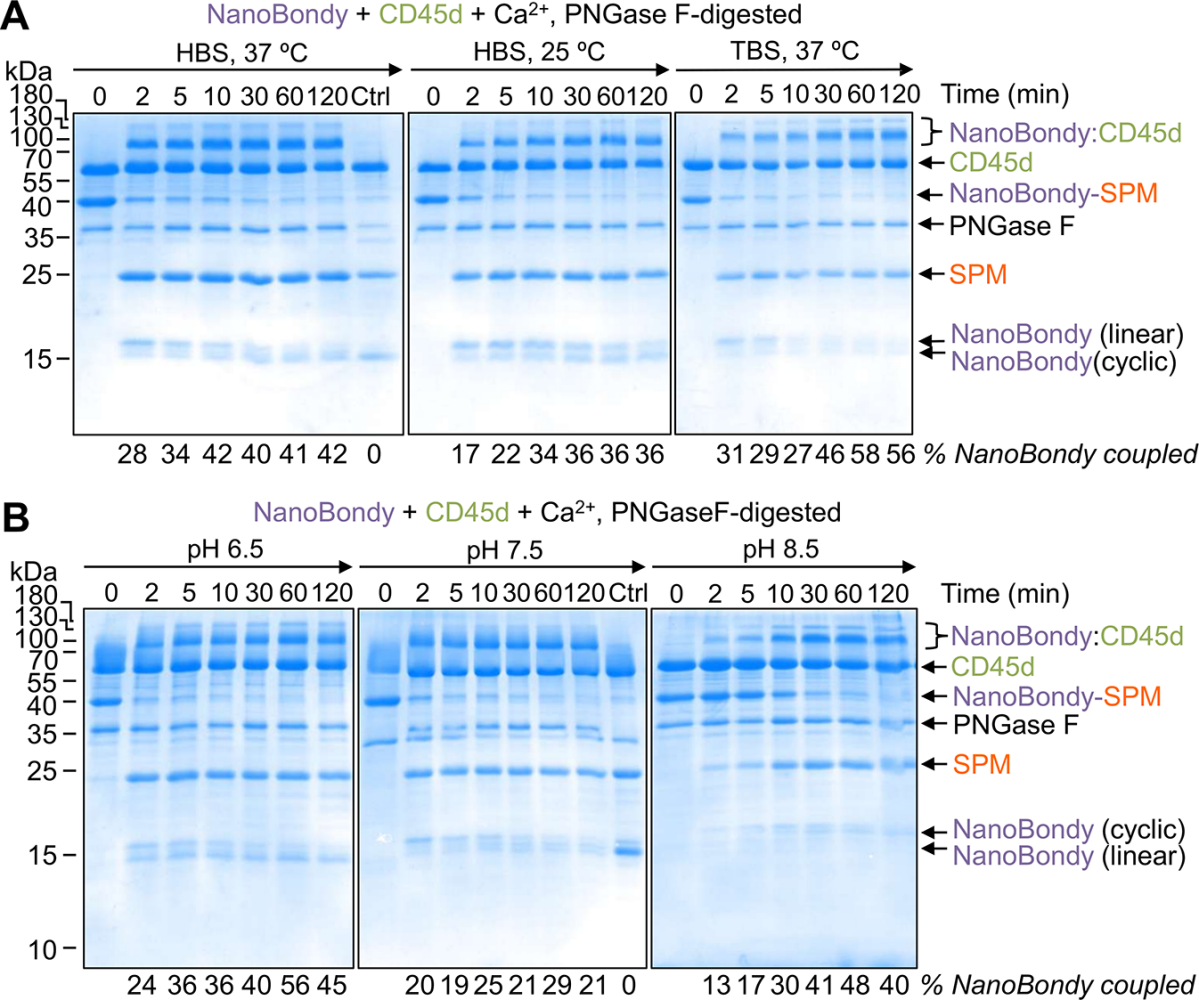
Condition-dependence of NanoBondy reaction. (A) Buffer- and temperature-dependence of NanoBondy reaction. 2H5 R72C anti-CD45 NanoBondy was incubated with CD45d, each at 10.5 μM, in the indicated buffer and temperature before SDS-PAGE with Coomassie staining. Ctrl refers to the lane containing anti-IgG NanoBondy with CD45d for 120 min. Representative gel from two independent experiments. (B) pH-dependence of NanoBondy reaction. The reaction was evaluated as in (A) with HBS-MES buffer at the indicated pH at 37 °C. Ctrl refers to the lane containing anti-IgG NanoBondy with CD45d for 120 min. The experiment was conducted once.

### Cross-Linking MS/MS Identifies Sites of NanoBondy-CD45d Cross-Linking

To identify the site of NanoBondy cross-linking to CD45d, the 2H5 R72C anti-CD45 NanoBondy and CD45d were mixed at 10.5 μM each in HBS, for a total protein content of 1 mg per reaction, before the addition of calcium. The cross-linked proteins were separated by high pH reverse-phase separation, and the fractions were analyzed by cross-linking tandem mass spectrometry (CL-MS/MS) to identify the dominant reaction sites (Figure 6). Cross-linking MS/MS indicated that the NanoBondy aspartyl anhydride formed covalent bonds predominantly to 4 different lysines on CD45d (Figures 6A/B and S4). The cross-linked lysines were all located near the AlphaFold-predicted interface, consistent with the structure prediction (Figure 6C).

**Figure 6:**
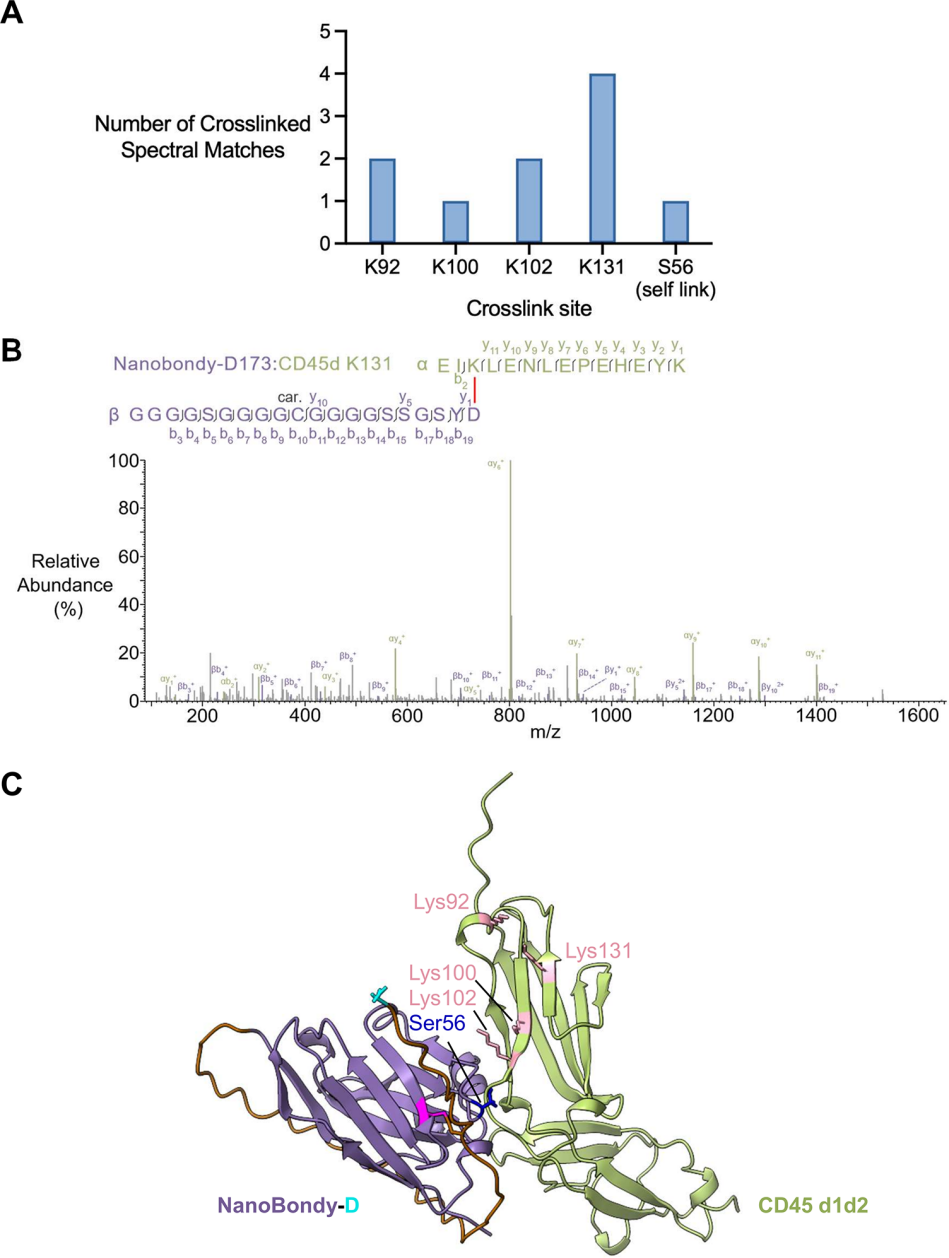
Mass spectrometry analysis of covalent conjugate between the NanoBondy and CD45d. (A) Dominant cross-linking sites. 2H5 R72C anti-CD45 NanoBondy was incubated with CD45d before cross-linking MS/MS. The number of identified cross-linked spectral matches for each NanoBondy cross-linking site on CD45 is shown. (B) Higher energy collision-induced dissociation (HCD) fragmentation spectrum of identified cross-link precursor ions corresponding to D173 (NanoBondy) to K131 (CD45d). Fragment ions matching fragmented cross-link (with cross-linker still intact) are annotated in bold, while peaks corresponding to fragments post-cross-link fragmentation are annotated with nonbold lines. “Car” indicates carbamidomethylation of cysteine. (C) Mapping of cross-link sites. AlphaFold prediction of NanoBondy (purple) bound to CD45d1d2 domains (green), highlighting cross-linking sites identified from the reactive aspartate (cyan) of the NanoBondy to target lysines (pink) or to serine (dark blue). Samples were run in technical triplicate. The cross-linking MS experiment was conducted twice.

To further investigate these predictions, we generated point mutations of CD45d at the predicted interface with NanoBondy (Figure S5A). The single mutants of CD45d, I104R or E105R, expressed well in Expi293F cells but caused a substantial loss in both covalent coupling by the anti-CD45 NanoBondy (Figure S5B) and noncovalent docking, as tested by ELISA (Figure S5C). These mutational experiments further validated the AlphaFold-predicted interface.

Cross-linking MS/MS identified a single own-goal site, where the NanoBondy anhydride formed an ester bond with Ser56 on the NanoBondy itself (Figure 6A/B, S4D). This is the first time that NeissLock has demonstrated covalent reaction with a serine.^40^ We have previously shown that the SPM anhydride is reactive to nucleophiles resembling the side chains of cysteine and tyrosine, as well as to α-amines, such as those at the protein N-terminus.^40^ The anti-CD45 NanoBondy contains two cysteine residues and 13 tyrosine residues. CD45d contains ten cysteine residues and six tyrosine residues. However, we did not observe NanoBondy-mediated conjugation to cysteine, tyrosine, or the α-amino group on either CD45d or the NanoBondy itself.

### NanoBondy Demonstrates Targeted Covalent Coupling at the Cell Surface

To test the NanoBondy’s reaction to CD45 at the cell surface, we initially used YTS, a human NK cell line. Cells were incubated with NanoBondy in the presence of 2 mM CaCl_2_ for 1 h at 37 °C. We evaluated NanoBondy reactivity to the YTS cell surface via Western blotting. When blotting for the nanobody moiety (VHH), we consistently observed a high molecular weight band following calcium addition, corresponding to the reaction product between NanoBondy and CD45. CD45RO migrates at 180 kDa.^51,52^ Therefore, the mass of the NanoBondy:CD45 conjugate would have an expected molecular weight of approximately 198 kDa. The observed covalent conjugate band migrated between the 185 and 270 kDa markers. We did not observe nonspecific bands, indicating that NanoBondy reaction is targeted to CD45 without promiscuous reactivity to diverse cell surface proteins (Figure 7A).

**Figure 7:**
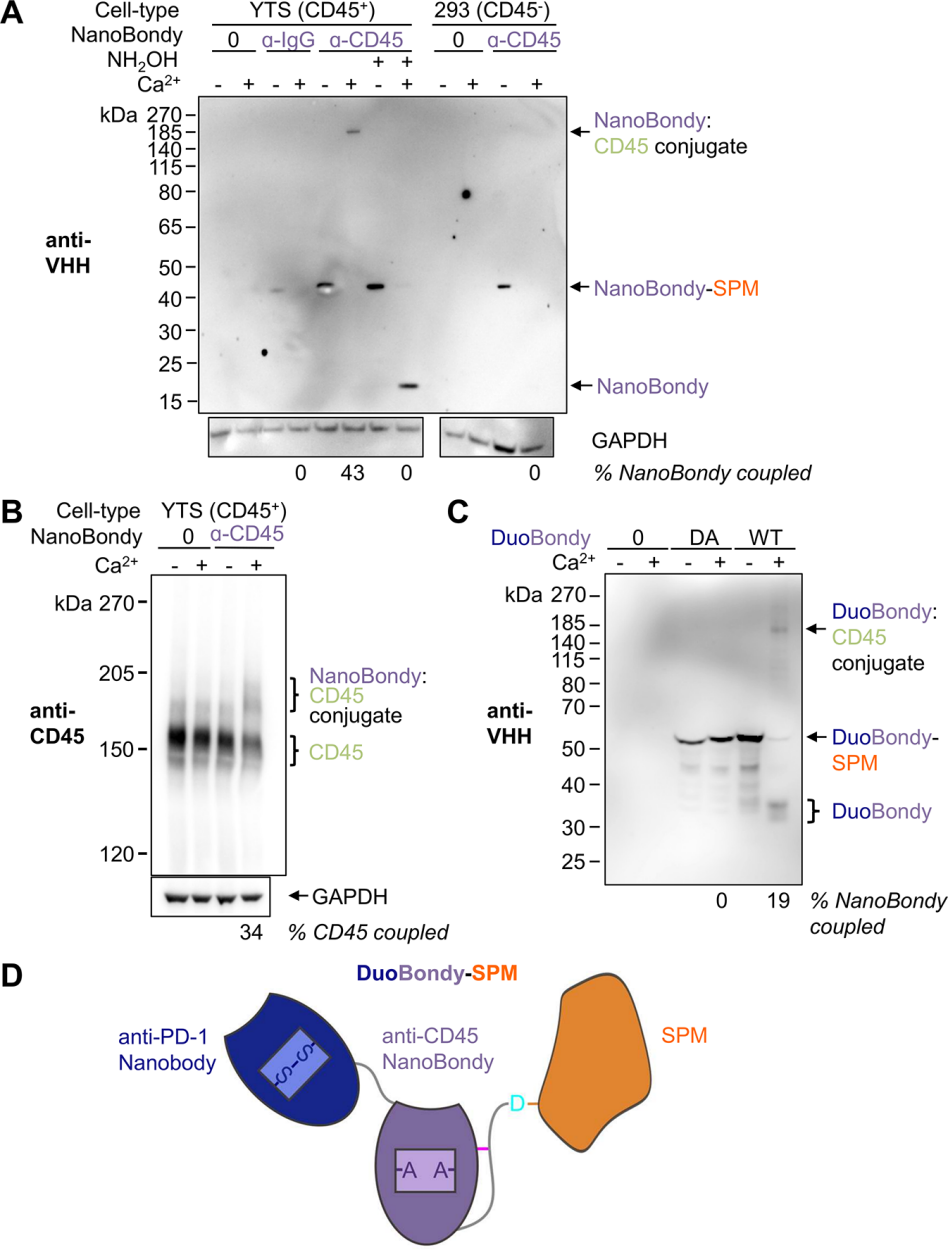
NanoBondy covalent conjugation at the cell surface. (A) Western blotting of NanoBondy reaction at the cell surface. Anti-CD45 NanoBondy at 5 μM was incubated with YTS cells or Expi293F cells for 1 h at 37 °C ± calcium. Covalent conjugation was evaluated by Western blot using an anti-VHH polyclonal antibody to detect the NanoBondy. Anti-IgG NanoBondy or hydroxylamine to react with the anhydride provided negative controls. Western blot to glyceraldehyde-3-phosphate dehydrogenase (GAPDH) was the loading control. The complete GAPDH blot is presented in Figure S6A. The experiment was conducted once. (B) YTS cells were stained as in (A), except with 25 μM anti-CD45 NanoBondy, and analyzed by Western blot using an anti-CD45 antibody. The CD45 band demonstrates an upward shift upon covalent conjugation with the a-CD45 NanoBondy. The full-length GAPDH blot is presented in Figure S6B. The experiment was conducted once. (C) Western blotting of the DuoBondy reaction on CD8^+^ T cells. DuoBondy (WT) or DuoBondy (DA) at 1 μM was incubated with CD8^+^ T cells for 40 min at 37 °C ± calcium. Covalent conjugation was evaluated by Western blot using an anti-VHH polyclonal antibody to detect the DuoBondy. Representative blot from two independent experiments. (D) DuoBondy consists of a nanobody binder (Nb102c3) to PD-1 (dark blue) fused N-terminally to the established covalently reacting anti-CD45 NanoBondy (purple) with SPM in orange.

Conjugation was blocked upon the addition of hydroxylamine, indicating that the observed product depends on anhydride-mediated reactivity. At 5 μM NanoBondy, we did not observe conjugation of the irrelevant NanoBondy control to CD45^+^ cells or conjugation of the anti-CD45 NanoBondy to the CD45^–^ Expi293F cells (Figure 7A). When blotting for CD45, we observed that the CD45 band demonstrated an upward shift upon NanoBondy reaction (Figure 7B), supporting that the NanoBondy is reacting with endogenous CD45 at the cell surface. To further test the cell-surface staining by the anti-CD45 NanoBondy, we incubated human primary CD8^+^ T cells (CD45^+^) or Expi293F cells (CD45^–^) and visualized cell-surface staining using live-cell confocal microscopy (Figure S8). Surface staining by the anti-CD45 NanoBondy was detected only on the CD8^+^ T cells.

To establish NanoBondy technology for covalent delivery of effector proteins, we generated a DuoBondy consisting of an anti-PD-1 nanobody^53^ attached N-terminally to the covalently reactive anti-CD45 NanoBondy (Figure 7C/D).^54^ In addition to the standard (WT) DuoBondy, we also generated a DA variant, where the reactive aspartate residue of SPM is mutated to alanine. This DA variant is therefore capable of noncovalent binding but not calcium-mediated cleavage or conjugation. To test the DuoBondy reaction, human primary CD8^+^ T cells were isolated from leukocyte blood cones. CD8^+^ T cells were incubated with 1 μM DuoBondy or DuoBondy DA in the presence of calcium. We evaluated the reactivity of DuoBondy to the CD8^+^ cell surface via Western blotting. The expected mass of the DuoBondy-D:CD45 conjugate is 217 kDa. When blotting for VHH, we consistently observed high molecular weight products following calcium addition that migrated from approximately 250 kDa to just below the 185 kDa marker, consistent with the reaction product between DuoBondy and CD45 (Figure 7C/D). We did not observe nonspecific bands, indicating that the DuoBondy reaction did not show promiscuous reactivity to other cell-surface proteins (Figure 7C). We observed conjugation only for the DuoBondy, with no covalent adduct detected for DuoBondy DA. These results demonstrate that NanoBondy technology allows covalent attachment of effector proteins to primary human cells and that fusion of an effector protein to the NanoBondy does not interfere with covalent cell conjugation.

## Discussion

Here, we have established NanoBondies, reengineering nanobodies for covalent reactivity through the inducible anhydride generation of NeissLock. We upgraded two different nanobodies to form covalent bonds with CD45. The reaction was compatible with different buffers and temperatures and showed specificity at the surface of an NK cell line and primary human CD8^+^ T cells. Previous use of NeissLock depended on the existence of a high-resolution structure in the Protein Data Bank to guide the reaction.^40^ Despite advances in computational structure prediction, there is still uncertainty in the prediction of protein binding interfaces, particularly for contacts through antibodies and nanobodies, where there are flexible loops and no evolutionary conservation.^55,56^ This work demonstrates the harnessing of protein binders for covalent coupling, even where there is no experimental structure.

Binders can be upgraded to covalent reactivity by incorporating weak electrophiles into binding interfaces, either through direct chemical coupling^57^ or unnatural amino acid mutagenesis.^33,58^ Initial electrophiles, such as acrylamides, were highly effective for reacting with exposed cysteines at the protein–protein interface but inefficient with other side chains.^57^ More recent use of SuFEx couples to a range of side chains, including lysine and tyrosine.^33,59^ However, the lack of inducible reactivity may pose challenges for the sustained storage of such reagents. Similar chemical or genetic routes may also be taken to attach photo-cross-linkers onto binders, but ultraviolet light activation of reactivity is damaging to cell survival and effector function.^60,61^ NeissLock reactivity is selectively induced under gentle conditions by adding extracellular levels of Ca^2+^, and the components are fully genetically encoded, using only canonical amino acids, which promotes simple and scalable production. Efficient NeissLock-driven protein:protein conjugation has been demonstrated for various protein pairs. Factors such as target protein glycosylation or the availability of surface lysines for conjugation may impact the overall yield of NeissLock-mediated conjugation. We demonstrate that reducing the strength of the initial noncovalent interaction between the NanoBondy and CD45d through the introduction of point mutations in CD45d decreases the subsequent degree of conjugation, supporting the idea that the strength of the initial noncovalent protein/protein interaction is important for the efficiency of reaction.

Our quantitation of cross-link frequency to different sites on the target provides valuable insights into the reach and residue preference of the anhydride from the NanoBondy. Previous MS on anhydride reactivity from NeissLock only identified a dominant cross-link to a lysine,^40^ but here we have quantified reactions to multiple lysine targets as well as a serine. This diverse range of possible sites for NeissLock coupling on the target is exciting, indicating that most proteins should be susceptible to ligation. Conversely, broad anhydride reactivity poses challenges in avoiding “own-goal” reaction sites on the NanoBondy itself when the highest reaction yield is desired. A limitation of NanoBondies is that competition between hydrolysis and coupling means this bioconjugation is unlikely to achieve near-quantitative target ligation, as may be achieved with SpyTag/SpyCatcher or HaloTag.^17,62^

Currently, our NanoBondy optimization pipeline involves testing a small set of different clamp locations, linker lengths, and own-goal lysine sites before validating the NanoBondy with the best expression, binding specificity, and reaction yield. Nanobodies or the related Sybodies are available for more than a thousand cellular targets,^63^ so the NanoBondy strategy has the potential to be generalized in diverse biological contexts. Despite internal disulfide bonds in nanobodies, we achieved efficient clamping through a novel disulfide bond in NanoBondies. In many cases, the core disulfide in the nanobody was not necessary for folding and expression^64^ and the clamp disulfide was well-formed in regular *E. coli* strains, not requiring strains optimized for an oxidizing cytosol.^65^ Most binding scaffolds (e.g., antibodies, affibodies, and DARPins)^66^ are like nanobodies in having the C-terminus away from the ligand-binding site, so it may be feasible in future work to use clamping to engineer these other platforms into NeissLock-based covalent binders.

CD45 represents an attractive initial target for covalent cell coupling because of its high expression on a wide range of hematopoietic cells and its stable surface expression.^14^ Future work may explore anti-CD45 NanoBondies on other cell types, given the cancer-targeting potential of CAR-macrophages and CAR-neutrophils.^67^ Future covalent targeting will also be valuable on red blood cells, which circulate for months and lack turnover of their plasma membrane.^35^ Beyond cell therapy, in biomaterials,^68^ biotransformation,^69^ gene therapy,^70^ and diagnostics,^71^ target-specific irreversible coupling with NanoBondies may enable new opportunities for molecular tenacity.

## Supplementary Material



## Notes

Comprehensive methods and supplementary results, including amino acid sequence, MS, structure prediction, and fluorescence microscopy (PDF)

## ∇

Institute of Molecular and Cell Biology, Agency for Science, Technology and Research (A*STAR), 61 Biopolis Drive, Singapore 138673, Singapore
