# Supplementary material for: NanoBondy Reaction through NeissLock Anhydride Allows Covalent Immune Cell Decoration
Source: Bioconjug Chem. 2026 Jan 24;37(2):281–92. doi: 10.1021/acs.bioconjchem.5c00519 (PMC12921663; doi:10.1021/acs.bioconjchem.5c00519)
Supplement: Supplementary file 1 [file bc5c00519_si_001.pdf]

Supplementary Methods

NanoBondy Reaction through NeissLock Anhydride Allows Covalent Immune Cell Decoration

Lasya R. Vankayala[1,2,3,4], Kish R. Adoni[5,6], Sheryl Y.T. Lim[1,7], Tommy Dam[4], Omer Dushek[4], Konstantinos Thalassinos[5,6], and Mark R. Howarth[2,3]*

[1]Department of Biochemistry, University of Oxford, South Parks Road, Oxford, OX1 3QU, UK.
[2]Department of Pharmacology, University of Cambridge, Tennis Court Road, Cambridge, CB2 1PD, UK.
[3]Engineering Biology Interdisciplinary Research Centre, University of Cambridge, Cambridge, CB2 1GA, UK.
[4]Sir William Dunn School of Pathology, University of Oxford, South Parks Road, Oxford, OX1 3RE, UK.
[5]Institute of Structural and Molecular Biology, Division of Biosciences, University College London, London, WC1E 6BT, UK.
[6]Institute of Structural and Molecular Biology, Birkbeck College, University of London, London, WC1E 6BT, UK.
[7]Current address: Institute of Molecular and Cell Biology, Agency for Science, Technology and Research (A*STAR), 61 Biopolis Drive, Singapore 138673, Singapore.

*Corresponding author:
Mark Howarth,
Department of Pharmacology,
University of Cambridge,
Tennis Court Road,
Cambridge,
CB2 1PD,
UK
E-mail: mh2186@cam.ac.uk

Plasmids and cloning

PCR was conducted using Q5 High-Fidelity 2× Master Mix (New England Biolabs). DNA primers and gBlock gene fragments were ordered from Integrated DNA Technologies. All open reading frames were verified by Sanger sequencing (Source Bioscience and Azenta). The CD45 nanobody sequences were as described[72]. 1E4, 2H5, 2F8, 1G4 and 1G1 gBlock gene fragments were cloned into pET28a containing a C-terminal SpyTag003 (RGVPHIVMVDAYKRYK)[73] using Gibson assembly, yielding pET28a-1E4-SpyTag003 (GenBank accession no. PX392308), pET28a 2H5-SpyTag003 (GenBank accession no. PX392309), pET28a-2F8-SpyTag003 (GenBank accession no. PX392310), pET28a-1G1-SpyTag003 (GenBank accession no. PX392311) and pET28a-1G4-SpyTag003 (GenBank accession no. PX392312). pET28a-NanoBondy 2H5(R72C) (GenBank accession no. PX392314, Addgene plasmid ID 247051) contains the anti-CD45 nanobody 2H5 with mutations C22A, C96A, R72C, K76R, followed by a flexible linker containing SpyTag003 (9 residues between the cysteine clamp and the reactive aspartate), then the sequence for full-length FrpA SPM and a C-tag[74]. Variants were made based on pET28a-NanoBondy 2H5(R72C) with altered clamp sites: K76C (GenBank accession no. PX392313) and G118C

(GenBank accession no. PX392315). The anti-CD45 2H5 R72C NanoBondy-His_6 variant was made based on pET28a-NanoBondy 2H5(R72C) with a His_6-tag in place of the C-tag. FrpA SPM consists of amino acids 298-543 of FrpA from *Neisseria meningitidis* serotype C (UniProt: P55126). 2F8 NanoBondy (C22A, C96A, N74C) (GenBank accession no. PX392316) was generated by Gibson assembly from pET28a-2F8(C22A, C96A)-SpyTag003. For generation of the anti-mouse IgG NanoBondy, the nanobody TP1170[75] with C22A and C96A mutations was ordered as a gBlock and cloned into pET28a-NanoBondy 2H5(R72C) with TP1170(C22A, C96A) replacing 2H5(C22A, C96A, R72C). pET28a TP1170 K98C IgG NanoBondy variant (GenBank accession no. PX392317) was used for subsequent experimentation.

pET28a-NanoBondy 2H5(R72C) was used as the parental construct for all 2H5 NanoBondy linker variants. Linker variants were named according to the number of residues between the cysteine clamp and the reactive aspartate. To clone linker variants of the 2H5 NanoBondy, the linker following SpyTag003 (GGGGSGGGGCGGGGSSGSY, 9 residue) was modified via Gibson Assembly to yield the following: GGGGSGGGGCGSY (3 residue), GGGGSGGGGCGSSGSY (6 residue), GGGGSGGGGCGGGGSSGASGSY (12 residue), and GGGGSGGGGCGGGGSSGASGAGSGSGSY (18 residue).

For the anti-PD-1 DuoBondy, a gBlock gene fragment of the anti-PD-1 nanobody Nb102c3[76] was ordered from Integrated DNA Technologies, then inserted N-terminal to the NanoBondy sequence using Gibson assembly. Nb102c3 and the NanoBondy sequence were separated by a GGGGSSGSYGSY linker. The DuoBondy expression vector is pET28a containing a C-terminal His_6. We further modified the DuoBondy sequence by replacing the loop-internal SpyTag003 with a GS linker of equivalent length. We then generated anti-PD-1 DuoBondy (DA) by mutating the reactive aspartate of the FrpA sequence to an alanine. Nanobodies, NanoBondies and DuoBondies were cloned in competent *E. coli* DH5α cells. pLEXm-hCD45d1d2 was a kind gift from Simon Davis, University of Oxford[77]. pLEXm-hCD45d1d2-AviTag was cloned by inserting the sequence for AviTag C-terminally to the coding sequence for domains 1 and 2 of human CD45, using restriction enzyme cloning. Complementary oligonucleotides encoding the AviTag sequence were annealed at equimolar concentration, then phosphorylated by T4 polynucleotide kinase (New England Biolabs). The hCD45d1d2 vector was also digested using KpnI and XhoI and was simultaneously incubated with Shrimp Alkaline Phosphatase (rSAP). Enzymes were heat-killed at 80 ºC for 20 min and digestion products were purified using a Monarch PCR and DNA cleanup kit (New England Biolabs). Insert and vector fragments were ligated using T4 DNA ligase (New England Biolabs) for 16 h at 18 ºC. pLEXm-hCD45d (GenBank accession no. PX392318, Addgene plasmid ID 247052), pLEXm-hCD45d(I104R) and pLEXm-hCD45d(E105R) were cloned by inserting the sequence for MBP C-terminal to the hCD45d1d2 (WT), hCD45d1d2(I104R) or hCD45d1d2(E105R) sequence via restriction enzyme cloning. The MBP gene was amplified via PCR, using primers to insert *KpnI* and *XhoI* sites. The MBP PCR product was purified using a Monarch PCR and DNA cleanup kit (New England Biolabs), then digested using KpnI and XhoI (New England Biolabs). The pLEXm-hCD45d1d2 vector was also digested using KpnI and XhoI and was simultaneously incubated with rSAP. Enzymes were heat-killed at 80 ºC for 20 min. Insert and vector fragments were ligated using T4 DNA ligase (New England Biolabs) for 16 h at 18 ºC. CD45d was cloned in New England Biolab Turbo cells.

For mouse CD45d1d2 (mCD45d1d2), a gBlock gene fragment of residues 203-375 of the mouse CD45 sequence was ordered from Integrated DNA Technologies. The mCD45d1d2 gene fragment was inserted by Gibson Assembly into a pcDNA3.1 vector downstream of a tPA signal sequence with a C-terminal His_6 tag for purification and AviTag for site-specific biotinylation. mCD45d1d2 was cloned in New England Biolab Turbo cells.

pET28a-His6-ODC-Ctag was previously described[78] and consists of human ODC1 (UniProt P11926) with an N-terminal His_6 tag and a C-terminal C-tag, cloned in pET28a (GenBank MW364944, Addgene plasmid ID 163614). pET28a-SpyTag003-sfGFP was also previously described[73] (Addgene plasmid ID 133454). SpyCatcher002-MBP was previously described[79] (GenBank MF974389 and Addgene plasmid ID 102831) and consists of SpyCatcher002 fused N-terminally to MBP in pET28. pET28a-nanoHER2-SpyTag003-His6 (GenBank accession no. PP341234, Addgene plasmid ID 216312) was previously described[79].

Bacterial Expression

Nanobodies, NanoBondies, sfGFP and ODC were transformed into competent *E. coli* BL21 DE3 cells (Agilent). Transformed cells were grown on LB Agar plates with 50 µg/mL kanamycin for 16 h at 37 ºC. A single colony was picked from the plate and used to inoculate 10 mL LB medium containing 50 µg/mL kanamycin. This starter culture was incubated at 37 ºC with shaking at 200 rpm for 16 h or until turbid (4 h). The starter culture was then used to inoculate 1 L of LB with 50 µg/mL kanamycin. The 1 L culture was incubated at 37 ºC with 200 rpm shaking. When OD_{600} reached 0.5-0.6, expression was induced with 0.42 mM isopropyl β-D-1-thiogalactopyranoside (IPTG) for 16 h at 18 ºC with 200 rpm shaking. Bacteria were pelleted by centrifugation at 4,000 g.

Mammalian Expression

mCD45d1d2, CD45d1d2, CD45d and CD45d mutants were expressed in Expi293F cells (Gibco). Expi293F cells were grown in Expi293F media (Gibco) under 80% humidity and 8% (v/v) CO_2 at 37 ºC at 95 rpm. Transfections were conducted according to manufacturer's recommendations using the Expi293F Transfection kit (Gibco). Briefly, Expi293F cells were brought to 3.0×10^6 cells/mL. 1 µg of plasmid DNA per mL of cell culture was incubated with Expifectamine reagent for 20 min at 25 °C. This plasmid-liposome mixture was then added dropwise to the prepared Expi293F cells, and the cell culture was returned to the incubator. 18-22 h following transfection, Expifectamine Transfection Enhancers 1 and 2 were added to the cell flask. On day 5 following enhancer addition, cells were pelleted by centrifugation at 300 g, and the supernatant was collected. The supernatant was spun again at 4,000 g, then sequentially syringe-filtered through 0.45 µm and 0.22 µm filters (Starlab).

C-tag purification

NanoBondies and ODC bait protein were purified via C-tag affinity purification. One pellet corresponding to 1 L of bacterial culture was resuspended in lysis buffer: 20 mM Tris-HCl, 150 mM NaCl at pH 7.4, supplemented with 4 mM ethylene glycol-bis(β-aminoethyl ether)-N,N,N′,N′-tetraacetic acid (EGTA), 0.1 mg/mL lysozyme, 1 mg/mL cOmplete mini EDTA-free protease inhibitor (Sigma-Aldrich) and 1 mM phenylmethanesulfonyl fluoride (PMSF). The resuspended pellet was then sonicated on ice for 1 min at 30% amplitude, with pulses of 1 s on and 1 s off. The pellet was allowed to rest on ice for 1 min, then the sonication and rest step were repeated 3 more times. The lysate was then spun at 30,000 g and the cleared lysate was collected. An Econo-PAC Chromatography Column (Bio-Rad) containing 1 mL of Capture Select C-tag XL Affinity Matrix (Thermo Fisher Scientific) was equilibrated by 2×10 column volume (CV) washes in C-tag wash buffer (20 mM Tris-HCl, 150 mM NaCl at pH 7.4). Cleared lysate was added to the column with equilibrated resin. Lysate was incubated with resin for 1 h at 4 ºC with end-over-end rotation. The resin was then washed twice with wash buffer (20 mM Tris-HCl, 150 mM NaCl, 4 mM EGTA, pH 7.4). Protein was eluted using 6 × 1 CV washes of elution buffer: 20 mM Tris-HCl, 1 M NaCl, 50% (v/v) propylene glycol and 2 mM EGTA. The amount of protein was evaluated by A_{280}. Extinction coefficients were based on ExPASy ProtParam[80].

Pooled elutions were dialyzed in 3.5 kDa molecular weight cut-off Spectra/Por tubing (Spectrum Labs) overnight at 4 ºC at 1,000-fold excess against TBS (20 mM Tris-HCl, 150 mM NaCl, pH 7.4) with 100 µM EGTA. This dialysis was repeated once more for 3 h. The pooled elutions were then dialyzed for a further 3 h against a 1,000-fold excess of HBS (50 mM HEPES, 150 mM NaCl, pH 7.4) with 100 µM EGTA. Dialyzed protein was concentrated using a 10 kDa molecular weight cut-off spin concentrator (Vivaspin). Protein was stored in aliquots at -80 ºC to minimize freeze-thawing. Protein concentrations were determined using A_{280}. Typical yield was 1 mg per L of culture for NanoBondies.

SpySwitch purification of SpyTag003-tagged nanobodies

Nanobody cell pellets were prepared as above but were resuspended in 1× SpySwitch buffer (50 mM Tris-HCl pH 7.5, 300 mM NaCl). The SpySwitch purification protocol has been described[81]. Briefly, SpySwitch resin (0.75-1 mL per 1 L bacterial culture) was equilibrated with 2×10 CV SpySwitch buffer in an Econo-PAC Chromatography Column (Bio-Rad). Clarified cell lysate was added to the equilibrated resin and incubated for 1 h at 4 ºC with end-over-end rotation. Supernatant was allowed to flow through by gravity. The resin was washed with 2× 10 CV SpySwitch buffer. For nanobody purification, pH-dependent elution was employed. 6× 1.5 CV of SpySwitch pH elution buffer (50 mM acetic acid, 150 mM NaCl, pH 5.0) was incubated with the resin for 5 min per elution. Elutions were collected into neutralization buffer (0.3 CV of 1 M Tris-HCl pH 8.0). Proteins were dialyzed as above, into TBS. Approximate yield for nanobodies was 1-2 mg per L of culture.

Ni-NTA purification

CD45d1d2, CD45d (CD45d1d2-MBP), CD45d binding site mutants, SpyTag003-sfGFP and SpyCatcher002-MBP were purified by nickel-nitrilotriacetic acid (Ni-NTA) affinity chromatography. Mammalian cell supernatant was collected and prepared as outlined above. The supernatants were supplemented with 10× Ni-NTA buffer (500 mM Tris-HCl, 3 M NaCl, pH 7.8) at 10% (v/v). 1 mL of Ni-NTA agarose (Qiagen) was packed in an Econo-PAC Chromatography Column (Bio-Rad). The packed resin was equilibrated with 2 × 10 CV of Ni-NTA buffer (50 mM Tris-HCl, 300 mM NaCl, pH 7.8). Prepared mammalian supernatant was then added to the equilibrated resin and incubated for 1 h at 4 ºC with end-over-end rotation. Supernatant was allowed to flow through by gravity. The resin was then washed with 2× 10 CV of Ni-NTA wash buffer (10 mM imidazole in Ni-NTA buffer). Protein was eluted by 6× 1 CV of elution buffer (200 mM imidazole in Ni-NTA buffer). Protein was dialyzed as described for NanoBondies, twice with TBS and once with HBS. Approximate yield for CD45d is 88 mg per L culture. Approximate yield for hCD45d1d2 is 13 mg per L culture. hCD45d1d2 was biotinylated as described[82].

NanoBondy-His_6 and DuoBondies were purified using cOmplete His-Tag purification resin (Roche). Bacterial cell pellets were lysed and prepared as above but were resuspended in sodium phosphate buffer (50 mM NaH_2PO_4, 300 mM NaCl, pH 8.0) supplemented with 2 mM EGTA and 5 mM imidazole. 1 mL cOmplete His-Tag agarose (Roche) was packed in an Econo-PAC Chromatography Column (Bio-Rad). The packed resin was then equilibrated with 2× 10 CV of sodium phosphate buffer. Prepared bacterial lysates were added to the equilibrated resin and incubated for 1 h at 4 ºC with end-over-end rotation. Supernatant was allowed to flow through by gravity. The resin was washed with 2× 10 CV of wash buffer (50 mM NaH_2PO_4, 300 mM NaCl, 10 mM imidazole, pH 8.0). Protein was eluted by 6× 1 CV elution buffer (50 mM NaH_2PO_4, 300 mM NaCl, 250 mM imidazole, pH 8.0). Protein was dialyzed as described above twice in TBS and once in HBS. Approximate yield for His_6-tagged NanoBondy was 7 mg and for DuoBondies was 1.5-4 mg per L of culture.

Polyacrylamide gel electrophoresis

For protein visualization, samples were mixed with 6× SDS loading buffer [234 mM Tris-HCl pH 6.8, 24% (v/v) glycerol, 120 µM bromophenol blue, 234 mM SDS] and 50 mM dithiothreitol (DTT) and heated at 99 °C for 3 min. Unless otherwise specified, SDS-PAGE was performed using 12-16% (w/v) polyacrylamide gels at 180 V in SDS-PAGE buffer [25 mM Tris-HCl, 192 mM glycine, 0.1% (w/v) SDS] in either an XCell SureLock system (Thermo Fisher) or a Mini Gel Tank (Invitrogen). Gels were washed twice with distilled water, then stained with Brilliant Blue G-250. Gels were destained in distilled water and imaged using an iBright FL1500 imaging system (Thermo Fisher), with analysis using iBright Analysis software versions 5.01 or 5.2.0 (Thermo Fisher).

Size-exclusion Chromatography

C-tag or His-Tag purified proteins were injected onto a pre-equilibrated Hi-Load 16/600 Superdex 200 pg column (GE Healthcare). Samples were run on an ÄKTA Pure 25 (GE Healthcare) fast protein liquid chromatography machine at 4 ºC into HBS + 100 µM EGTA, pH 7.4. Elutions were monitored by absorbance at 230 nm, 260 nm and 280 nm. Peak analysis was conducted using SDS-PAGE and desired fractions were concentrated at 4 ºC using a 10 kDa molecular weight cut-off spin concentrator (Vivaspin). Proteins were stored at -80 ºC.

PNGase F expression and digestion

pOPH6, a kind gift from Shaun Lott (Addgene plasmid ID 40315)[83],was transformed into competent *E. coli* BL21 DE3, as described above, and grown on LB Agar plates with 100 µg/mL carbenicillin for 16 h at 37 ºC. Bacterial growth, induction and expression were conducted as above. A bacterial pellet corresponding to 1 L of culture was resuspended in 50 mL ice-cold periplasmic lysis solution (0.5 M sucrose, 0.1 M Tris-HCl, 1 mM EDTA, pH 8.0), then pelleted again at 3,000 g for 20 min and the supernatant discarded. The pellet was then resuspended in 50 mL ice-cold MilliQ water and incubated for 10 min on ice. To the resuspended pellet was added $MgCl_2$ to a final 1 mM and the mixture was incubated on ice for a further 10 min. The bacteria were pelleted again at 3,000 g for 20 min and the supernatant retained. This supernatant was supplemented with 10× Ni-NTA buffer (500 mM Tris-HCl, 3 M NaCl, pH 7.8) at 10% (v/v). PNGase F was purified using Ni-NTA as described above. Size-exclusion chromatography was used as a secondary purification step as above. PNGase F was obtained at ~11 mg per L of culture. Purified PNGase F was aliquoted and stored at -20 ºC in HBS + 20% (v/v) glycerol.

For digestion, to the reaction mixture was added 10× glycoprotein denaturing buffer (New England Biolabs) at 10% (v/v). Reaction was heated at 100 ºC for 10 min, then centrifuged using a benchtop mini centrifuge at 25 ºC and 2,000 g for 1 min, and cooled on ice for 2 min. To this denatured reaction was added 10× glycoprotein buffer 2 (New England Biolabs) and 10% (v/v) NP-40 (New England Biolabs), both at a final 10% (v/v). MilliQ H_2O was added to reach the desired final volume. Finally, 1 µg of PNGase F was added to each reaction. Digestion was conducted at 37 ºC for 2 h.

ELISA

Nunc MaxiSorp plates (Thermo Fisher) were coated with 80 nM SpyCatcher002-MBP in PBS (137 mM NaCl, 2.7 mM KCl, 10 mM Na_2HPO_4 and 1.8 mM KH_2PO_4, pH 7.4) and incubated for 16 h at 4 ºC. Plates were washed 3 times with TBS-T [TBS + 0.1% (v/v) Tween-20], with a 5 min incubation for each wash. Plates were blocked for 1 h at 25 °C in blocking buffer: 1% (w/v) bovine serum albumin (BSA) (Sigma-Aldrich) in TBS-T. SpyTag003-fused nanobodies were added to the blocked plates at a final 80 nM in TBS and incubated for 1 h at 25 °C. Plates were washed 3 times with TBS-T for 5 min per wash. CD45d1d2-biotin was added to the plates

in 10-fold serial dilutions in TBS and incubated for 1 h at 25 °C. Plates were washed 3 more times with TBS-T, incubating for 5 min per wash. Pierce High Sensitivity Streptavidin-horseradish peroxidase (HRP) (Thermo Fisher) diluted to 0.2 μg/mL in blocking buffer was added to the plates and incubated for 1 h at 25 °C. The plates were washed 6 times with TBS-T, with 5 min per wash. Plates were incubated at 25 ºC with 1-step Ultra TMB-ELISA Substrate Solution (Thermo Scientific). The reaction was stopped at the 2 min time-point by the addition of 1 M HCl.

For ELISA in Figure S5C, a Nunc MaxiSorp plate was coated with 80 nM of CD45d variants (WT, E105R or I104R) in PBS and incubated for 16 h at 4 ºC. Plates were washed 3 times with TBS-T, with a 5 min incubation for each wash. Plates were blocked for 1 h at 25 °C in blocking buffer: 1% (w/v) BSA (Sigma-Aldrich) in TBS-T. 2H5 R72C NanoBondy was added to the plates in 10-fold serial dilutions in TBS and incubated for 1 h at 25 °C. Plates were washed 3 more times with TBS-T, incubating 5 min per wash. MonoRab anti-VHH HRP (GenScript, A01861) diluted to 0.2 μg/mL in blocking buffer was added to the plates and incubated for 1 h at 25 °C. The plates were washed 6 times with TBS-T, with 5 min per wash. Plates were incubated at 25 ºC with 1-step Ultra TMB-ELISA Substrate Solution (Thermo Fisher). The reaction was stopped at the 2 min time-point by the addition of 1 M HCl.

Absorbance measurements were collected at 450 nm (A_{450}) using a FLUOstar Omega plate reader (BMG Labtech) and Omega MARS software. Binding curves were visualized by GraphPad PRISM software (version 10.1.1).

Cell culture for immortalized cell lines

Expi293F cells were from Thermo Fisher. YTS cells (RRID:CVCL_D324) and NK92 cells (RRID:CVCL_2142)[84] were a kind gift from Daniel Davis (Imperial College London). Expi293F cells were grown in Expi293 media (Thermo Fisher) at 37 ºC, 80% humidity and 8% (v/v) CO_2 with shaking according to manufacturer's guidelines. YTS cells were grown in Roswell Park Memorial Institute 1640 (RPMI-1640) supplemented with 10% (v/v) fetal bovine serum (FBS), 2 mM L-glutamine, 10 mM HEPES, 1 mM sodium pyruvate, 4.5 g/L glucose and 1.5 g/L sodium bicarbonate. NK92 cells were grown in Minimum Essential Medium Alpha (MEM-Alpha) supplemented with 10% (v/v) FBS, 10% (v/v) horse serum, 50 mM 2-mercaptoethanol, 2 mM GlutaMAX, and 200 U/mL rhIL-2 (PeproTech). YTS and NK92 cells were grown at 37 ºC and 5% (v/v) CO_2. Cells were passaged at 70-80% confluency. Cells were sub-cultured for less than 3 months. Cells were validated as mycoplasma-negative by PCR.

Flow Cytometry for Nanobody binding

YTS, NK92 and Expi293F cells were washed in PBS and seeded at 1 million cells/well of a 96 well CELLSTAR V-bottom plate (Greiner). Cells were incubated with 2 μM purified nanobody or 1 μg/mL anti-human CD45 antibody (Invitrogen, clone HI30) in flow buffer [PBS + 1% (w/v) BSA] for 1 h at 25 °C. Cells were washed 3 times in PBS by centrifugation at 150 g at 4 ºC for 3 min. Cells were then incubated with secondary antibody in flow buffer for 1 h at 25 °C. For samples incubated with mouse anti-human CD45, the secondary antibody was polyclonal Alexa Fluor 647-conjugated, highly cross-adsorbed goat anti-mouse IgG (Invitrogen, RRID AB_2535805), at 4 μg/mL in flow buffer at 25 ºC. For samples incubated with nanobody, the secondary antibody was iFluor647-conjugated MonoRab rabbit anti-camelid VHH antibody (GenScript) at 1 μg/mL in flow buffer at 25 ºC. Cells were washed 4 times at 4 ºC. Cells were then resuspended in LIVE/DEAD Fixable Aqua (Invitrogen) at 1 μL reconstituted stain per 1 million cells and incubated for 30 min at 25 °C in the dark. Cells were washed with PBS, then resuspended in PBS. To the resuspended cells was added an equal volume of 4% (w/v) paraformaldehyde in PBS, for a final concentration of 2% (w/v) for 20 min at 25 ºC. To these fixed cells was added PBS. Cells were then centrifuged, washed once

in flow buffer, then resuspended in fresh flow cytometry buffer at 25 ºC. Samples were immediately analyzed on a CytoFlex LX analyzer. Compensation was conducted at the start of each analysis. Data were collected using CytExpert version 2.5 and analyzed using FlowJo version 10.1.0. Events were gated on cells, singlets, then live cells prior to binding analysis.

Protein conjugation assays

Reactions were carried out in HBS at 37 °C. In cases of hydroxylamine block, reactions were carried out in HBS + 15 mM hydroxylamine (Sigma-Aldrich) at 37 °C. NanoBondy and CD45d were mixed in HBS at 10.5 μM each. Reaction was started by addition of 2 mM $CaCl_2$, with 2 mM EGTA pH 8.0 added to 'no calcium' controls. Reactions were incubated for 1 h, then quenched by addition of 10× glycoprotein denaturing buffer (New England Biolabs) at 10% (v/v). Reactions were PNGase F-digested as described above, then visualized by SDS-PAGE with Coomassie staining. Crosslinking between proteins at different sites leads to different polypeptide chain organizations with distinct gel mobility, as previously shown[78].

Protein conjugation time-courses

Unless otherwise specified, reactions were carried out in HBS pH 7.4 at 37 °C. To measure pH-dependence, HBS was supplemented with 50 mM MES to buffer across the desired pH range. To measure buffer-dependence, reactions were also carried out in TBS pH 7.4 at 37 °C. For both pH- and buffer-dependency experiments, proteins were buffer-exchanged into the desired buffer using a Vivaspin 500 10 kDa MWCO spin concentrator (Vivaspin) prior to assembling reactions.

A reaction mix was assembled by adding NanoBondy and CD45d at 10.5 μM in the desired buffer. Reaction was held at the desired temperature and initiated with 2 mM $CaCl_2$. At each timepoint, a fraction of the reaction mix was removed and quenched by addition of 4× stop buffer (60 mM EGTA and 4× glycoprotein denaturing buffer) for a final EGTA concentration of 15 mM and final protein concentrations of 10 μM. Quenched reactions were immediately heated at 100 °C for 10 min. For the 0 min timepoint, stop buffer was added first, followed by 2 mM $CaCl_2$. Reactions were PNGase F-digested as described above, then visualized by SDS-PAGE with Coomassie staining. Protein concentration following PNGase F-digestion was 5 μM.

Quantification of protein conjugation using gel densitometry

Quantification of band intensity for SDS-PAGE was conducted using iBright Analysis software (Thermo Fisher, version 5.4.0) with rolling ball correction. Relative intensity of each product band was calculated by the iBright software by determining the ratio of the intensity of the product band (e.g. NanoBondy:CD45d) to the intensity of the NanoBondy-SPM band from the calcium-free lane:

$$\text{Relative intensity}_{\text{NanoBondy:CD45d}} = \frac{\text{Intensity}_{\text{NanoBondy:CD45d}}}{\text{Intensity}_{\text{NanoBondy-S}}}$$

For lanes with multiple product bands, the relative intensity of each band was independently determined and then summed. To determine % NanoBondy coupled, molecular weight correction was applied:

$$\%\ \text{NanoBondy coupled} = \text{Relative intensity}_{\text{NanoBondy:CD45d}} \times \frac{\text{Molecular Weight}_{\text{NanoBondy-SP}}}{\text{Molecular Weight}_{\text{NanoBondy:CD45d}}} \times 100$$

For conditions where the software failed to detect a product band, % NanoBondy conjugated was set to 0.

Quantification of band intensity for Western blots in Figure 7A and 7B was conducted using iBright Analysis software (Thermo Fisher, version 5.4.0) with rolling ball correction. Quantification of band intensity for Figure 7C was conducted using Image Lab software (Bio-Rad, version 6.1.0). For Figures 7A and 7C, the uncleaved NanoBondy-SPM band was used

as the reference band. For Figure 7B, the CD45 band population without calcium addition was used as the reference band. For Figure 7B, band boundaries were manually set. For Figures 7A and 7C, bands were automatically detected. No molecular weight correction was performed for Western blot quantification, since detection was based on antibody recognition.

For Figures 7A and 7C, % NanoBondy coupled was calculated by determining the ratio of the intensity of the product band (NanoBondy:CD45) to the intensity of the NanoBondy-SPM band from the calcium-free lane:

$$\% \text{ NanoBondy coupled} = \frac{\text{Intensity}_{\text{NanoBondy:CD45}}}{\text{Intensity}_{\text{NanoBondy}-\text{SPM}}} \times 100$$

For Figure 7B, % CD45 conjugated was calculated by determining the ratio of the intensity of the product band (NanoBondy:CD45) to the intensity of the major CD45 band from the calcium-free lane.

$$\% \text{ CD45 coupled} = \frac{\text{Intensity}_{\text{NanoBondy:CD45}}}{\text{Intensity}_{\text{CD45}}} \times 100$$

Intact protein ESI Mass Spectrometry

For intact protein MS on anti-CD45 2H5 R72C NanoBondy-His_6, a RapidFire 365 platform (Agilent) was used. The Rapid365 platform consisted of a jet-stream electrospray ionization source coupled to a 6550 Accurate-Mass Quadrupole Time-of-Flight (Q-TOF) (Agilent). 5 µM of 2H5 NanoBondy in HBS was diluted 1:1 with water before formic acid was added to a final concentration of 0.9% (v/v). The protein sample was aspirated under vacuum for 0.3 s and loaded onto a C4 solid-phase extraction cartridge. Washes were conducted using 0.1% (v/v) formic acid in water for 5.5 s. The sample was then eluted onto the Q-TOF detector for 5.5 s. MassHunter Qualitative Analysis B.07.00 (Agilent) was used for data analysis. Expected protein molecular weight was calculated using the ExPASy ProtParam tool[80], with 2 Da subtracted to account for the formation of a disulfide bond[85].

Crosslinking MS sample preparation

The reaction mix was assembled by adding 2H5 R72C NanoBondy to CD45d in HBS to final protein concentrations of 10.5 µM NanoBondy and 10.5 µM CD45d. The total mass of protein in the reaction was 1 mg. Reaction was initiated with 2 mM $CaCl_2$ and held at 37 ºC for 2 h. Reaction was PNGase F-digested as described above. PNGase F was heat-inactivated by holding the reaction at 75 ºC for 10 min. Reaction mix was transferred to -20 ºC for overnight storage and transported to mass spectrometry analysis on dry ice.

An equal volume of 5% (w/v) SDS with 100 mM Tris-HCl (pH 8.5) was added to 500 µg calcium-cleaved 2H5 R72C NanoBondy:CD45d conjugate, before the sample was incubated at 99 ºC for 10 min, followed by water bath sonication (Fisherbrand, FB11203) at 37 kHz, power 100, at 25 ºC on sweep mode for a further 10 min. Ammonium bicarbonate (pH 8.5) was added to a final 50 mM, before addition of DTT to a final 8 mM, followed by incubation at 37 ºC for 30 min. Iodoacetamide was then added to a final 20 mM and incubated for 30 min in darkness at 25 ºC before adding trypsin (Trypsin Gold, Promega) at a ratio of 50:1 analyte:trypsin, followed by overnight incubation at 37 ºC with shaking. Digestion was quenched with a final trifluoroacetic acid concentration of 0.5% (v/v), before desalting using Waters Oasis HLB Solid Phase Extraction (SPE) plate. Samples were then reconstituted in Buffer A (100 µL 10 mM ammonium formate, pH 10.0), before fractionation (ACQUITY UPLC BEH C18 VanGuard Pre-column 130Å, 1.7 µm, 2.1 mm × 5 mm) using a gradient of 4% to 35% Buffer B [80% (v/v) acetonitrile, 10 mM ammonium formate, pH 10.0] over 8 min with fractions collected every 20 s. Fractions were then pooled to a total of 8 fractions and each fraction was reconstituted in 0.1% (v/v) formic acid before LC-MS/MS.

LC-MS/MS (Crosslinking Mass Spectrometry)

An UltiMate 3000 RSLCnano liquid chromatography system (Thermo Fisher), with 50 cm µPAC Neo HPLC analytical column and 0.075 mm × 20 mm trap cartridge (Acclaim PepMap C18 100 Å, 3 µm) was connected to an Orbitrap Eclipse Tribrid mass spectrometer (Thermo Fisher) via a SilicaTip emitter. Column temperature was set to 45 ºC, with an analytical column flow rate of 750 nL/min. Mobile phase A was 0.1% (v/v) formic acid with 3.2% (v/v) acetonitrile and mobile phase B was 0.1% (v/v) formic acid with 96.8% (v/v) acetonitrile. An elution gradient from 3% to 55% mobile phase B over 48 min was applied, with a total run time of 60 min. The Orbitrap Eclipse Tribrid mass spectrometer was externally calibrated using Pierce FlexMix calibration solution and nano-ESI was performed using a SilicaTip emitter, connected to the LC *via* an HPLC liquid junction tee.

Spray stability and signal intensity were optimized by varying the SilicaTip electrospray to a final positive ion voltage of 2,000 V and modifying the Silica Tip positioning in the *x*, *y* and *z* dimensions. Transfer capillary temperature was set to 275 ºC, RF lens was set to 40%, precursor ion mass resolution was set to 120,000, precursor ion mass range was set to 350 -2000 *m/z* and precursor ion charge state was set from 3^+ to 7^+. MS^1 spectra were acquired with a Data-Dependent Analysis duty cycle (Top20), automatic gain control (AGC) was set to target 400,000 (100%), maximum injection time mode was set to Auto, precursor ions were isolated with a 1.4 *m/z* window using a quadrupole mass filter, and monoisotopic precursor selection was set to peptide peak determination. For Orbitrap MS^2 acquisition using Higher energy Collision-induced Dissociation (HCD), collision energy was set to 30%, resolution was set to 30,000, mass range was set to normal, AGC was set to normal with an absolute AGC value of 5.00×10^4 and maximum injection time mode was set to Auto.

The samples were run in technical triplicate and RAW files were processed using Proteome Discoverer 2.5 (Thermo Fisher) with the Sequest HT[86] database searching node and the XlinkX[87] crosslink processing node. For Sequest HT, dynamic modifications included: oxidation (+15.995 Da, M) and N-terminal acetylation (+42.011 Da). Static modifications included: carbamidomethyl (+57.021 Da, C) with false discovery rate (FDR) set to 0.01 using the Target Decoy PSM Validator node. The FASTA file database contained sequences for 2H5 R72C NanoBondy, CD45d, PNGase F and 200 randomly selected decoy proteins for false discovery analysis. For crosslink identification, the FASTA file contained 2H5 R72C NanoBondy , CD45d and PNGase F. Relative to the FASTA file amino acid sequence, aspartic acid and lysine/threonine/serine/arginine/tyrosine/α-amino sites were considered to crosslink with an overall chemical difference of O(-1)H(-1) from the peptides off the initial proteins 2H5 R72C NanoBondy and CD45d to the crosslinked entity, corresponding to a zero length crosslinker average mass of -17.0073 Da and a monoisotopic mass of -17.00274 Da. Crosslink residues were selected as: D-K, D-T, D-S, D-R, D-Y and D-α-amino. XlinkX acquisition strategy was set to Noncleavable_fast and minimum signal/noise: 1.5. FDR was set to 0.05 in the XlinkX PD Validator node. Only high confidence crosslinks from D173 of 2H5 R72C NanoBondy were considered for further analysis and all identified crosslinks were manually verified from their corresponding MS/MS fragmentation spectra. The number of crosslinked spectral matches was reported by XlinkX, with the corresponding scan number used to manually annotate the MS/MS spectra from within the RAW file. All data are available on PRIDE (accession number PXD063277) and all validated crosslinks are provided in the Supplementary Information.

Western blotting

YTS cells were seeded at 1.5 million cells/well of a CELLSTAR 96 well V-bottom plate (Greiner). $CD8^+$ T cells were seeded in 15 mL falcon tubes at 4 million cells/condition. Cells were washed twice with HBS at 25 ºC. Washed cells were resuspended with 5 µM NanoBondy

in HBS with or without 5 mM hydroxylamine, or with 1 µM DuoBondy (WT) or DuoBondy (DA) in HBS + 1.5% (w/v) BSA. Reaction was initiated by addition of 2 mM $CaCl_2$, with 2 mM EGTA added to 'no calcium' controls. Cells were incubated at 37 °C for 1 h for NanoBondy assays or 37 ºC for 40 min for DuoBondy assays. Cells were then washed once in HBS at 25 ºC. For lysis, cells were resuspended in 180 µL/million cells ice-cold RIPA buffer [20 mM Tris-HCl, 150 mM NaCl, 1% (v/v) Triton X-100, 0.5% (w/v) sodium deoxycholate, 0.1% (w/v) SDS, pH 7.4] freshly supplemented with 1 mM PMSF and 1× cOmplete mini EDTA-free inhibitors (Roche). Resuspended cells were transferred to 1.5 mL microcentrifuge tubes and incubated on ice for 20 min. Lysed cells were then centrifuged at 12,000 g for 20 min at 4 °C to pellet nuclei. Lysate was mixed with 6× SDS loading buffer [234 mM Tris-HCl pH 6.8, 24% (v/v) glycerol, 120 µM bromophenol blue, 234 mM SDS] to a final volume of 1× SDS loading buffer. For anti-VHH visualization, 50 mM DTT was included, whereas for anti-CD45 visualization reducing agent was not used. Lysates were heated at 99 °C for 3 min before loading onto SDS-PAGE. For visualization of NanoBondy conjugation products, samples were loaded onto a 4-12% NuPAGE Bis-Tris gel (Invitrogen) and run in MOPS Buffer (Invitrogen) at 180 V for 1 h using a Mini Gel Tank (Invitrogen). For visualization of DuoBondy conjugation products, samples were loaded onto a 4-12% NuPAGE Bis-Tris gel (Invitrogen) and run in MES Buffer (Invitrogen) at 180 V for 1 h using an XCell SureLock Mini-Cell (Invitrogen). For visualization of CD45, samples were loaded onto a 6% Tris-Acetate gel and run in Tris-glycine buffer [25 mM Tris-HCl, 192 mM glycine, 0.1 % (w/v) SDS] at 180 V for 1 h using a Mini Gel Tank (Invitrogen). Gels were washed in MilliQ water, then soaked in 20% (v/v) ethanol in MilliQ water for 10-20 min with gentle rocking at 25 ºC. Proteins were transferred to Polyvinylidene fluoride (PVDF) membranes using an iBlot2 system (Invitrogen) and iBlot2 PVDF Transfer Stack (Invitrogen). Transfer was conducted at 15 V for 13 min.

For anti-VHH visualization, the blocking buffer was TBS with 5% (w/v) skimmed milk and 0.05% (v/v) Tween-20. For anti-CD45 and anti-GAPDH visualization, the blocking buffer was TBS with 3% (w/v) BSA and 0.05% (v/v) Tween-20. Membranes were blocked at 4 °C for 16 h. Blocked membranes were incubated with primary antibodies for 1 h at 25 °C; mouse anti-human CD45 (Invitrogen clone HI30) at 1 µg/mL or mouse anti-human GAPDH (antibodies.com, clone GA1R) at 2 µg/mL or AffiniPure goat anti-alpaca IgG VHH domain (Jackson ImmunoResearch, RRID AB_2810907) at 0.6 µg/mL. Membranes were washed 3 times with TBS + 0.05% (v/v) Tween-20, incubating 3 min per wash at 25 ºC. Membranes were then incubated with secondary stain for 1 h at 25 °C. For anti-CD45 and anti-GAPDH visualization, the secondary antibody was anti-mouse IgG peroxidase antibody (Sigma-Aldrich) at 1:2,500 in blocking buffer. For anti-VHH, the secondary antibody was rabbit anti-goat IgG HRP (Invitrogen, RRID AB_2534006) at 0.2 µg/mL dilution in blocking buffer. Membranes were then washed 3 times in TBS-T, incubating 3 min per wash at 25 ºC. Blots were visualized by SuperSignal West Pico PLUS Chemiluminescent Substrate (Thermo Fisher) using an iBright FL1500 imaging system (Thermo Fisher).

Primary CD8$^+$ T cell isolation and transduction

Human CD8$^+$ T cells were isolated from leukocyte cones using negative selection. Blood samples were incubated with 150 µL Rosette-Sep Human CD8$^+$ enrichment cocktail (STEMCELL Technologies) per mL of blood for 20 min at 25 ºC. The blood:enrichment cocktail mixture was diluted 2:1 with PBS and layered on top of Ficoll Paque Plus (GE) at a ratio of 0.8 Ficoll to 1.0 blood mixture. Falcon tubes containing the layered preparation were centrifuged at 1,200 g for 20 min at 25 ºC with slow acceleration and deceleration. The resulting buffy coat, containing isolated CD8$^+$ T cells, was collected. Cells were counted, then resuspended at 1 million cells/mL in complete RPMI: RPMI + 10% (v/v) FBS and 100 U/mL penicillin/streptomycin with 50 U/mL IL-2 (PeproTech) and CD3/CD28 human T-activator

DynaBeads (Thermo Fisher) at a 1:1 bead to cell ratio. Isolated $CD8^+$ human T cells were cultured at 37 ºC and 5% (v/v) CO_2.

Confocal microscopy to evaluate NanoBondy cell-surface labeling
Primary human $CD8^+$ T cells were isolated and expanded as in the previous section. $CD8^+$ T cells or Expi293F cells were washed once in HBS, then seeded at 200,000 cells/well of a CELLSTAR 96 well V-bottom plate (Greiner). Cells were stained with 2 µM anti-CD45 NanoBondy (2H5 R72C with 9 residue linker) in HBS with 1% (w/v) BSA and 100 µM EGTA for 1 h at 25 ºC. Cells were then washed 3 times in HBS. Secondary staining was conducted using Alexa Fluor 647-conjugated AffiniPure goat anti-alpaca IgG VHH domain (Jackson ImmunoResearch, RRID AB_2810907) at 0.6 µg/mL in HBS + 1% (w/v) BSA for 40 min at 25 ºC. Cells were then washed 4 times in HBS.

Cells were imaged live on a Zeiss 880 Airyscan laser scanning microscope using a Plan-Apochromat 63× oil immersion objective (Zeiss, NA 1.4) with a pinhole setting of 1 Airy Unit (AU) and a pixel size of 0.1318 µm/pixel. Image acquisitions were controlled by the Zen Black Software. In each region of interest an image was captured by using a solid-state laser with an excitation wavelength of 633 nm, operating with a laser power of 27 µW and a power density 148 mW/cm^2. For every region of interest, a brightfield image was also captured. All imaging was done with a line averaging of 2. The resulting image stacks were handled using Fiji (ImageJ version 1.54p)[88], and the image collage was assembled in Illustrator (Adobe version 29.7.1), presenting a confocal slice. Images for the different samples were collected and processed using the same settings.

Ethics declaration for use of human samples
Leukocyte cones were purchased from the National Health Service's (UK) Blood and Transplantation service (NHS-BT). All cones were anonymized by the NHS-BT before purchase. Ethical approval was provided by the Health Research Authority at National Health Service through a Research Ethics Committee (20/EM/0267) submitted through the Medical Sciences Inter-divisional Research Ethics Committee (IDREC) at the University of Oxford (R51997/RE001). Study samples were obtained by the authority of the institutional board that licensed the use of the material.

Data Analysis and Visualization
Data visualization for ELISA and flow cytometry activation curves was performed using GraphPad Prism 10 (GraphPad Software). Protein structures were visualized using PyMOL version 2.5.4 (Schrödinger). Figures for publication were generated using UCSF ChimeraX version 1.8rc202405230136[89]. NanoBondy structure and docking were predicted using AlphaFold with ColabFold version 1.5.2[90]. To generate AlphaFold2-multimer models of nanobody docking to the extracellular domain of human CD45, an amino acid sequence corresponding to PDB accession code 5FN7 (domains 1 and 2) was used[77]. Figure 2B depicts the CD45 RO isoform, which corresponds to the most abundant CD45 isoform on YTS[91] and activated $CD8^+$ T cells[92].

Safety/hazard statement
Hydroxylamine hydrochloride was handled according to all recommended safety precautions. Dilute solutions for experimental use were prepared within a chemical fume hood. No other unexpected or unusually high safety hazards were encountered during this study.

Data Availability Statement

Plasmids encoding the NanoBondy and CD45d constructs have been deposited in the Addgene repository (https://www.addgene.org/Omer_Dushek/) and sequences of other constructs have been deposited in GenBank as described in the section "Plasmids and cloning". For Crosslinking MS, all validated crosslinks are provided in the Supplementary Information. The MS data that support the findings of this study are openly available in PRIDE at https://www.ebi.ac.uk/pride/, reference number PXD063277. Any requests for further information, or for resources and reagents should be directed to and will be fulfilled by the lead contact, M.R.H..

References

(72) Rokkam, D.; Lupardus, P. J. *Discovery and Characterization of Llama VHH Targeting the RO Form of Human CD45*; preprint; bioRxiv, 2020; p 2020.09.01.278853. https://doi.org/10.1101/2020.09.01.278853.

(73) Keeble, A. H.; Turkki, P.; Stokes, S.; Khairil Anuar, I. N. A.; Rahikainen, R.; Hytönen, V. P.; Howarth, M. Approaching Infinite Affinity through Engineering of Peptide–Protein Interaction. *Proc. Natl. Acad. Sci. U.S.A.* **2019**, *116* (52), 26523–26533. https://doi.org/10.1073/pnas.1909653116.

(74) Huang, J.; Nagy, S. S.; Koide, A.; Rock, R. S.; Koide, S. A Peptide Tag System for Facile Purification and Single-Molecule Immobilization. *Biochemistry* **2009**, *48* (50), 11834–11836. https://doi.org/10.1021/bi901756n.

(75) Pleiner, T.; Bates, M.; Görlich, D. A Toolbox of Anti–Mouse and Anti–Rabbit IgG Secondary Nanobodies. *J. Cell Biol.* **2018**, *217* (3), 1143–1154. https://doi.org/10.1083/jcb.201709115.

(76) Kalinin, R. S.; Ukrainskaya, V. M.; Chumakov, S. P.; Moysenovich, A. M.; Tereshchuk, V. M.; Volkov, D. V.; Pershin, D. S.; Maksimov, E. G.; Zhang, H.; Maschan, M. A.; Rubtsov, Y. P.; Stepanov, A. V. Engineered Removal of PD-1 From the Surface of CD19 CAR-T Cells Results in Increased Activation and Diminished Survival. *Front. Mol. Biosci.* **2021**, *8*, 745286. https://doi.org/10.3389/fmolb.2021.745286.

(77) Chang, V. T.; Fernandes, R. A.; Ganzinger, K. A.; Lee, S. F.; Siebold, C.; McColl, J.; Jönsson, P.; Palayret, M.; Harlos, K.; Coles, C. H.; Jones, E. Y.; Lui, Y.; Huang, E.; Gilbert, R. J. C.; Klenerman, D.; Aricescu, A. R.; Davis, S. J. Initiation of T Cell Signaling by CD45 Segregation at "Close Contacts." *Nat Immunol* **2016**, *17* (5), 574–582. https://doi.org/10.1038/ni.3392.

(78) Scheu, A.; Lim, S. Y. T.; Metzner, F. J.; Mohammed, S.; Howarth, M. NeissLock Provides an Inducible Protein Anhydride for Covalent Targeting of Endogenous Proteins. *Nat Commun* **2021**, *12* (1), 717. https://doi.org/10.1038/s41467-021-20963-5.

(79) Driscoll, C. L.; Keeble, A. H.; Howarth, M. R. SpyMask Enables Combinatorial Assembly of Bispecific Binders. *Nat Commun* **2024**, *15* (1), 2403. https://doi.org/10.1038/s41467-024-46599-9.

(80) Gasteiger, E.; Hoogland, C.; Gattiker, A.; Duvaud, S.; Wilkins, M. R.; Appel, R. D.; Bairoch, A. Protein Identification and Analysis Tools on the Expasy Server. In *The Proteomics Protocols Handbook*; Humana Press, 2005; pp 571–607.

(81) Vester, S. K.; Rahikainen, R.; Khairil Anuar, I. N. A.; Hills, R. A.; Tan, T. K.; Howarth, M. SpySwitch Enables pH- or Heat-Responsive Capture and Release for Plug-and-Display Nanoassembly. *Nat Commun* **2022**, *13* (1), 3714. https://doi.org/10.1038/s41467-022-31193-8.

(82) Fairhead, M.; Howarth, M. Site-Specific Biotinylation of Purified Proteins Using BirA. *Methods Mol Biol* **2015**, *1266*, 171–184. https://doi.org/10.1007/978-1-4939-2272-7_12.

(83) Loo, T.; Patchett, M. L.; Norris, G. E.; Lott, J. S. Using Secretion to Solve a Solubility Problem: High-Yield Expression in Escherichia Coli and Purification of the Bacterial

Glycoamidase PNGase F. *Protein Expression Purif.* **2002**, *24* (1), 90–98. https://doi.org/10.1006/prep.2001.1555.
(84) Gong, J. H.; Maki, G.; Klingemann, H. G. Characterization of a Human Cell Line (NK-92) with Phenotypical and Functional Characteristics of Activated Natural Killer Cells. *Leukemia* **1994**, *8* (4), 652–658.
(85) Klapoetke, S.; Xie, M. H. Disulfide Bond Characterization of Human Factor Xa by Mass Spectrometry through Protein-Level Partial Reduction. *J. Pharm. Biomed. Anal.* **2017**, *132*, 238–246. https://doi.org/10.1016/j.jpba.2016.10.005.
(86) Brodbelt, J. S.; Russell, D. H. Focus on the 20-Year Anniversary of SEQUEST. *J. Am. Soc. Mass Spectrom.* **2015**, *26* (11), 1797–1798. https://doi.org/10.1007/s13361-015-1264-1.
(87) Liu, F.; Lössl, P.; Scheltema, R.; Viner, R.; Heck, A. J. R. Optimized Fragmentation Schemes and Data Analysis Strategies for Proteome-Wide Cross-Link Identification. *Nat Commun* **2017**, *8* (1), 15473. https://doi.org/10.1038/ncomms15473.
(88) Schneider, C. A.; Rasband, W. S.; Eliceiri, K. W. NIH Image to ImageJ: 25 Years of Image Analysis. *Nat Methods* **2012**, *9* (7), 671–675. https://doi.org/10.1038/nmeth.2089.
(89) Meng, E. C.; Goddard, T. D.; Pettersen, E. F.; Couch, G. S.; Pearson, Z. J.; Morris, J. H.; Ferrin, T. E. UCSF ChimeraX: Tools for Structure Building and Analysis. *Protein Sci.* **2023**, *32* (11), e4792. https://doi.org/10.1002/pro.4792.
(90) Mirdita, M.; Schütze, K.; Moriwaki, Y.; Heo, L.; Ovchinnikov, S.; Steinegger, M. ColabFold: Making Protein Folding Accessible to All. *Nat Methods* **2022**, *19* (6), 679–682. https://doi.org/10.1038/s41592-022-01488-1.
(91) Gunesch, J. T.; Angelo, L. S.; Mahapatra, S.; Deering, R. P.; Kowalko, J. E.; Sleiman, P.; Tobias, J. W.; Monaco-Shawver, L.; Orange, J. S.; Mace, E. M. Genome-Wide Analyses and Functional Profiling of Human NK Cell Lines. *Molecular Immunology* **2019**, *115*, 64–75. https://doi.org/10.1016/j.molimm.2018.07.015.
(92) Al Barashdi, M. A.; Ali, A.; McMullin, M. F.; Mills, K. Protein Tyrosine Phosphatase Receptor Type C (PTPRC or CD45). *J Clin Pathol* **2021**, *74* (9), 548–552. https://doi.org/10.1136/jclinpath-2020-206927.

anti-CD45 NanoBondy

MSGQVQLQESGGGLVQTGGSLTLSAVASGGTFSSYAMGWF 40

RQAPGKEREFVAAIGGSGDSTYYADSVKGRFTISCDNARN 80

SVYLQMNSLKPEDTAVYYAQADPTMFHKLYYGINPNEYDY 120

WGQGTLVTVSSGGGGSGGRGVPHIVMVDAYKRYKGGGGSG 160

GGGCGGGGSSGSYDPLALDLDGDGIETVAAKGFAGALFDH 200

RNQGIRTATGWVSADDGLLVRDLNGNGIIDNGAELFGDNT 240

KLADGSFAKHGYAALAELDSNGDNIINAADAAFQTLRVWQ 280

DLNQDGISQANELRTLEELGIQSLDLAYKDVNKNLGNGNT 320

LAQQGSYTKTDGTTAKMGDLLLAADNLHSRFKDKVELTAE 360

QAKAANLAGIGRLRDLREAAALSGDLANMLKAYSAAETKE 400

AQLALLDNLIHKWAETDGSSHHHHHH 426

anti-CD45 2H5 (C22A, C96A, R72C, K76R)
Clamp site
Linker
Reactive D
SPM
His_6

Supplementary Figure 1. **Amino acid sequence of lead anti-CD45 NanoBondy (2H5 R72C with 9 residue spacer from clamp site to reactive D).** Residue numbers begin at 1 from the N-terminal methionine, which is cleaved in the final protein sequence.

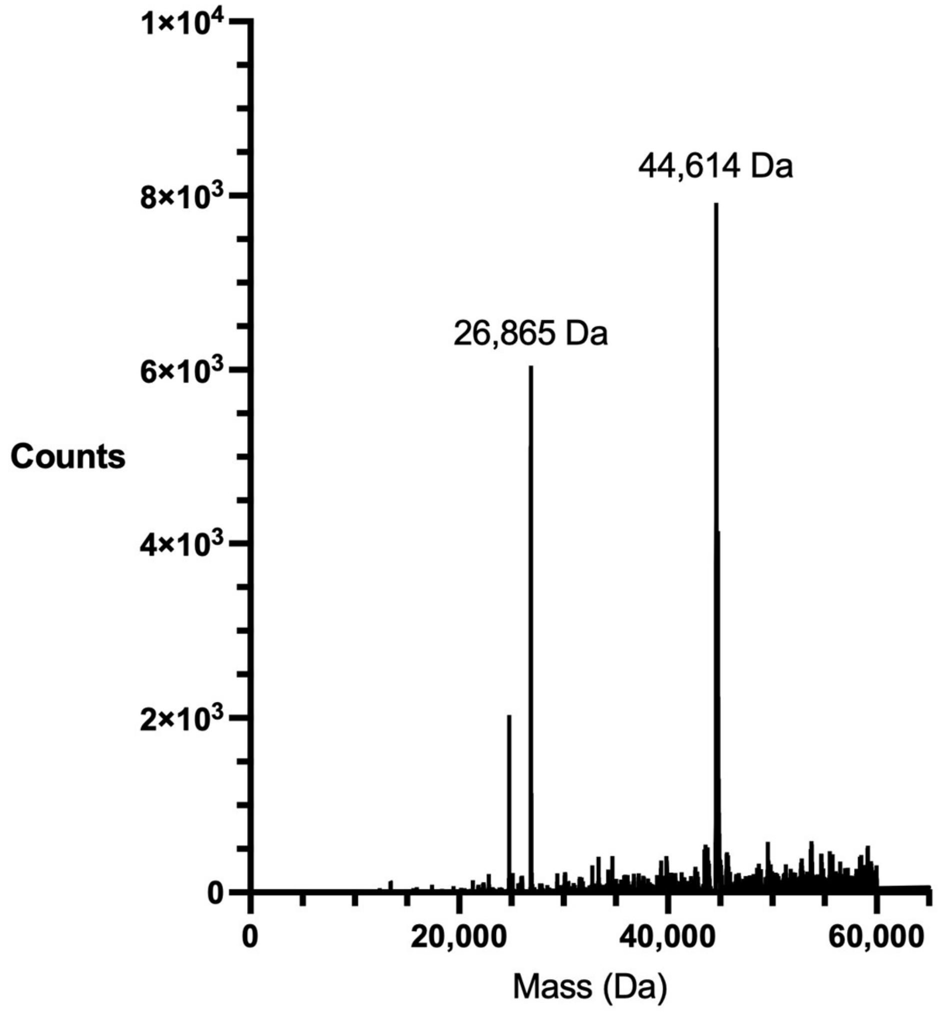

***Supplementary Figure 2*. Intact mass spectrometry of NanoBondy**. Electrospray ionization mass spectrometry of anti-CD45 2H5 R72C NanoBondy-His_6. Expected mass of the full-length NanoBondy after disulfide formation is 44,614 Da. Expected mass of the SPM cleavage product is 26,865 Da. Representative mass spectrum from two independent experiments.

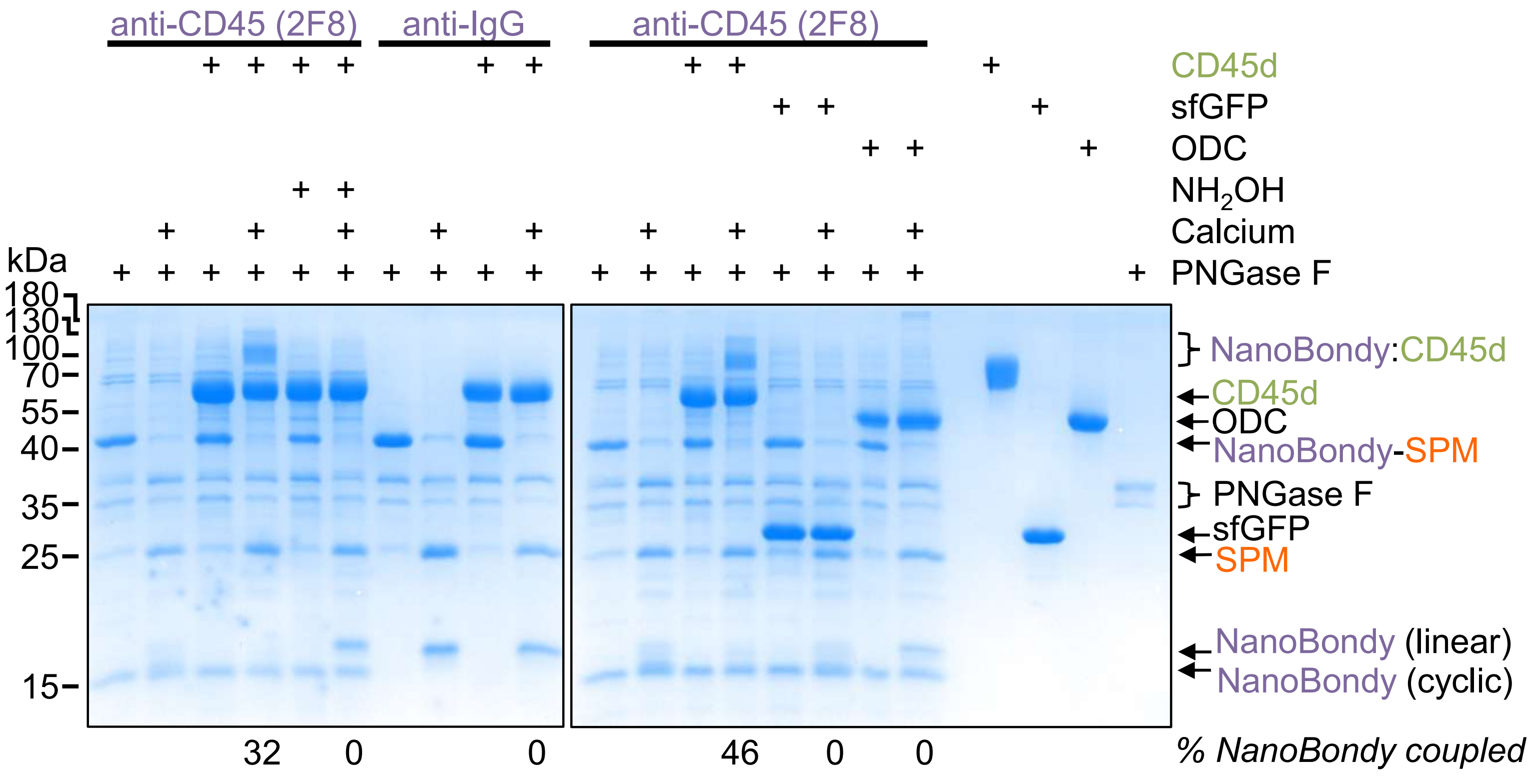

Supplementary Figure 3. **Specificity of 2F8-derived NanoBondy coupling to CD45**. α-CD45 2F8 NanoBondy was incubated ± calcium with CD45d or control proteins ornithine decarboxylase (ODC) or superfolder GFP (sfGFP) for 1 h at 37 ºC in HBS. Hydroxylamine was a competitor. α-IgG NanoBondy was a negative control. Formation of the covalent product was visualized using SDS-PAGE/Coomassie. The experiment was conducted once.

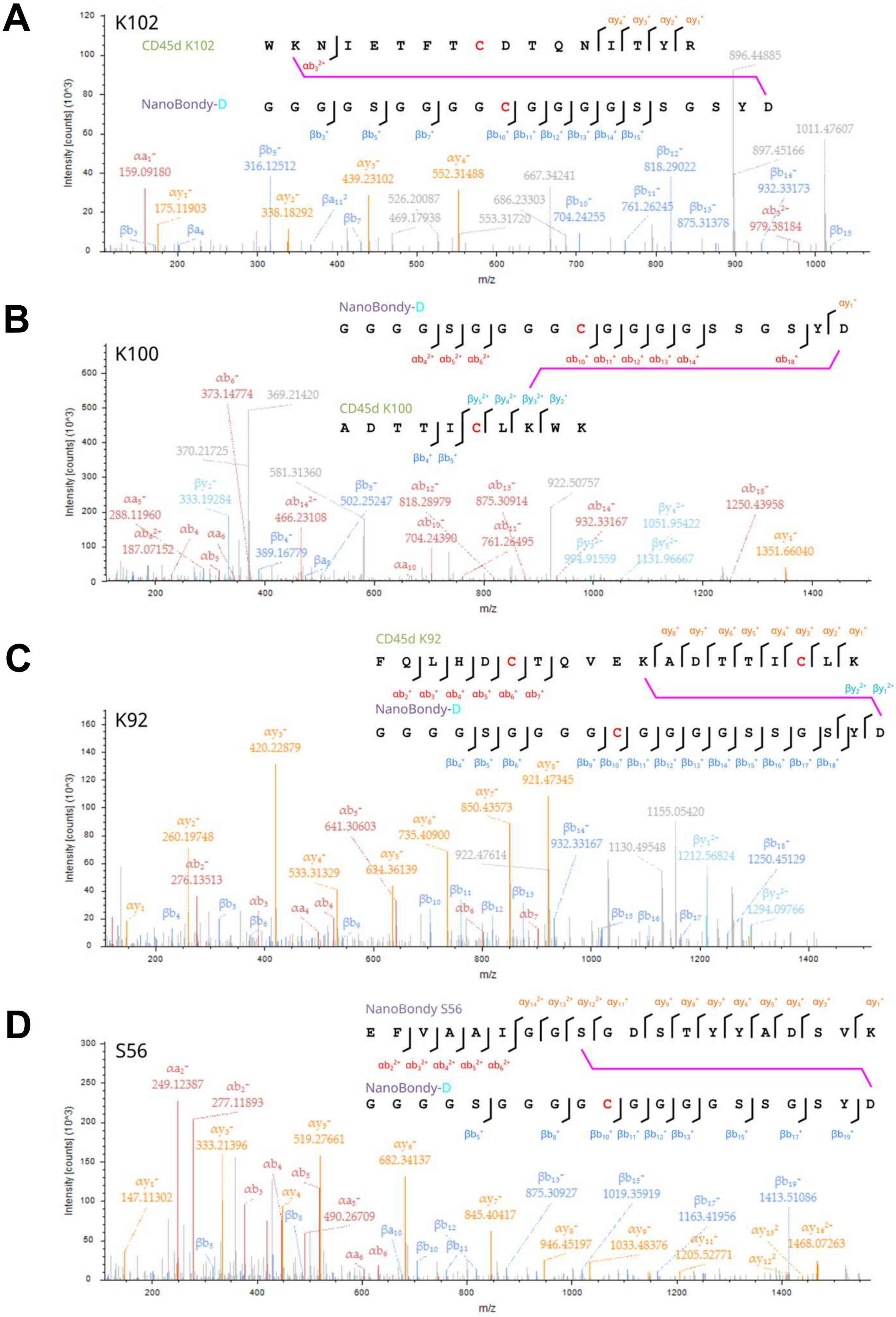

Supplementary Figure 4. **NanoBondy crosslinks mapped in MS/MS.** Higher energy collision-induced dissociation (HCD) fragmentation spectra of identified crosslink precursor ions corresponding to D173 (2H5 R72C NanoBondy) peptide coupled to a CD45d peptide: **(A)** K102, **(B)** K100, **(C)** K92, and self-link corresponding to NanoBondy D173 coupled to **(D)** NanoBondy S56. MS/MS spectra taken directly from Proteome Discoverer. Cysteines are colored red to indicate carbamidomethylation. The two peptides are depicted as α and β peptides (α peptide above β peptide in figure). α-b (red), α-y (orange), β-b (dark blue) and β-y (light blue) ions are marked. Some spectra also contain a ions, which were ignored for fragment annotation. Samples were run in technical triplicate. Deep fractionation MS was conducted once.

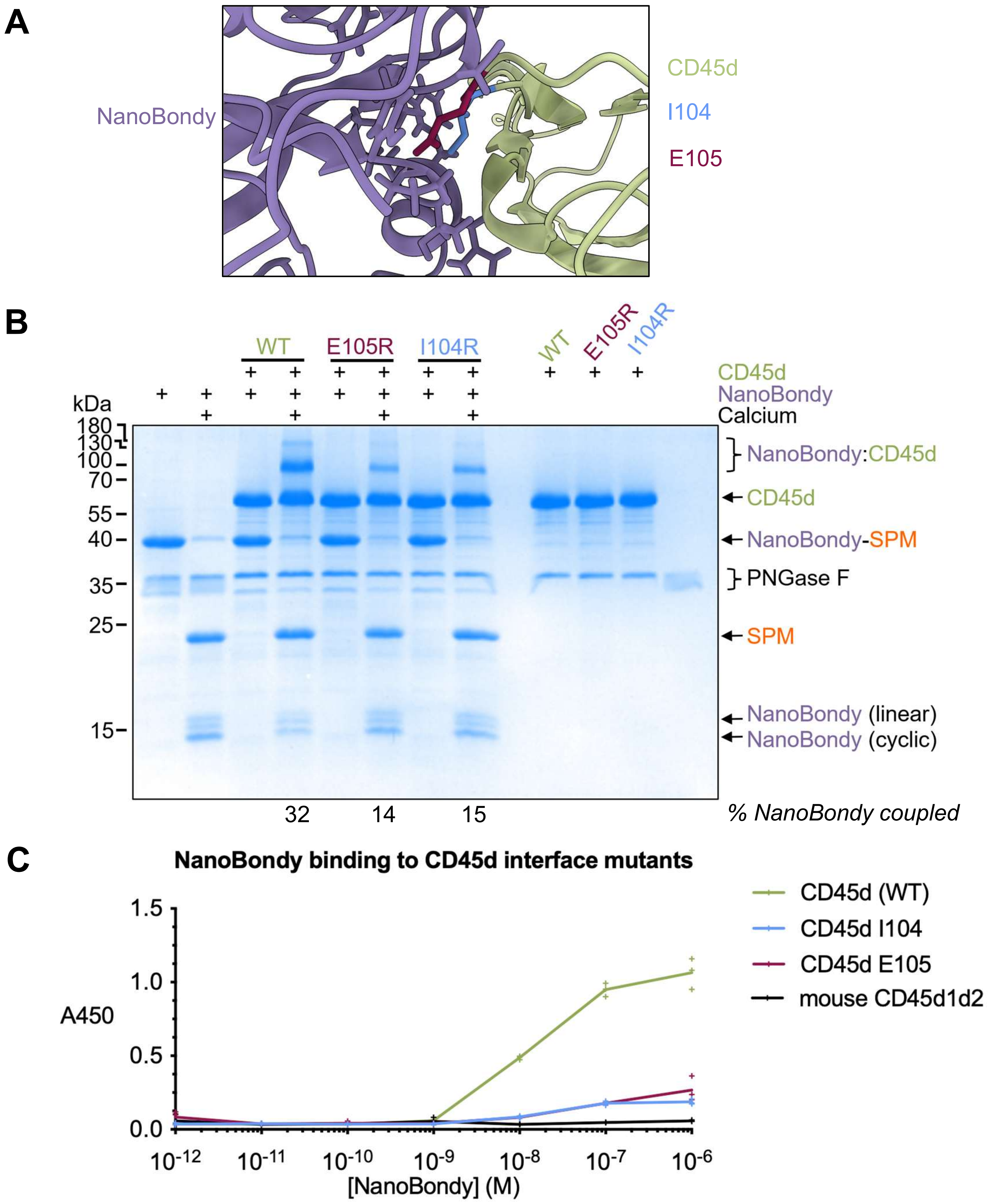

Supplementary Figure 5**. Validation of predicted NanoBondy docking site on CD45. (A)** AlphaFold2multimer prediction of 2H5 NanoBondy:CD45d docking. E105 (red) and I104 (blue) of CD45 sit at the docking interface. NanoBondy residues 33-36 and 103-107 shown in stick form. **(B)** 2H5 R72C NanoBondy conjugation to CD45d mutants was evaluated by SDS-PAGE/Coomassie. 10.5 µM NanoBondy was incubated with 10.5 µM CD45d variants for 1 h at 37 ºC ± Ca^{2+}, then digested with PNGase F for 2 h. PNGase F was included in all lanes. Representative gel from two independent experiments. **(C)** NanoBondy binding to CD45d mutants evaluated using ELISA. WT CD45d, CD45d mutants, or mouse CD45d (mCD45d1d2, negative control) were bound to the plate and incubated with a titration of NanoBondy variants. NanoBondy binding was detected using anti-VHH HRP. Each triplicate data point is shown, with a line connecting the mean. ELISA was conducted once.

A

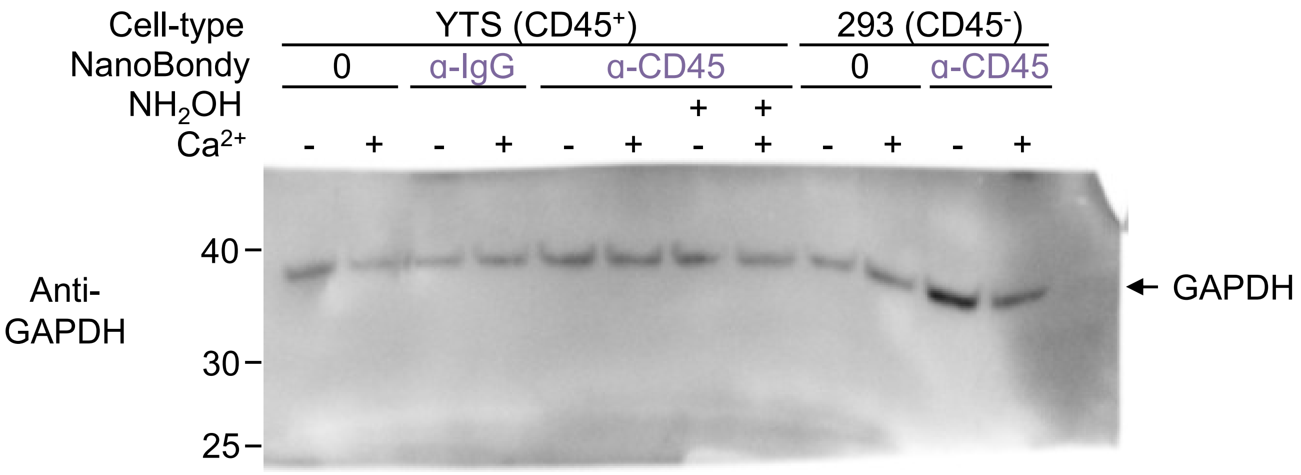

B

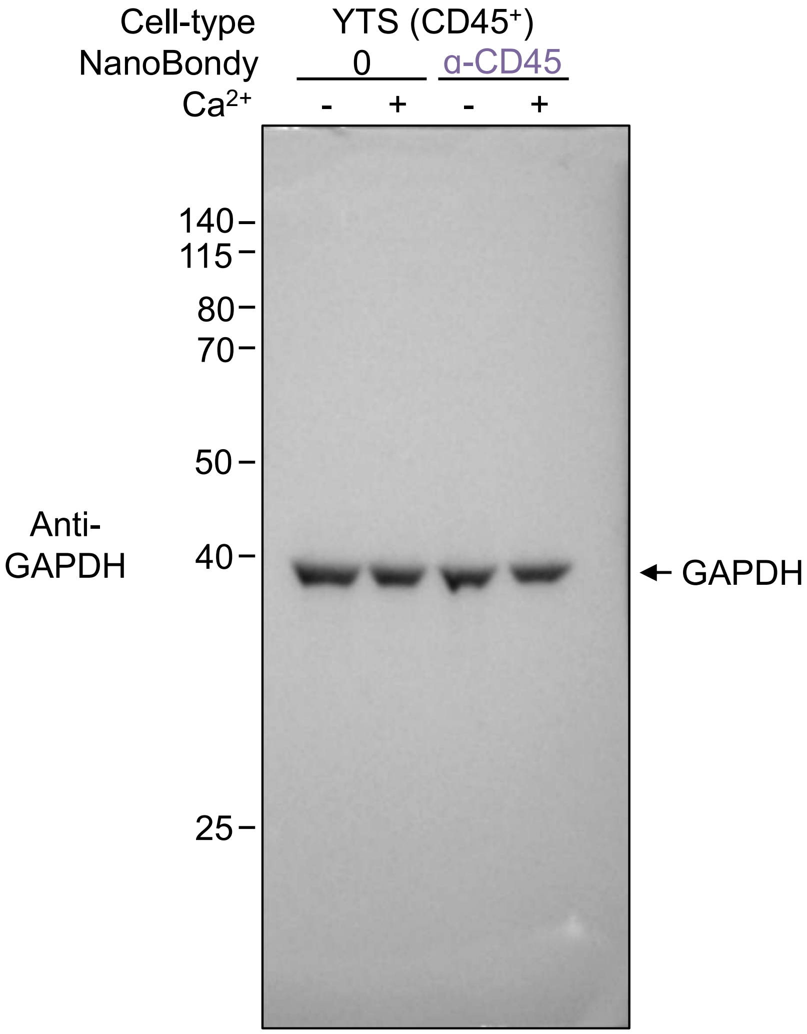

***Supplementary Figure 6*. Full-size GAPDH blots to accompany Figure 7. (A)** Western blotting of NanoBondy reaction. 5 μM anti-CD45 NanoBondy was incubated with YTS cells or Expi293F cells for 1 h at 37 ºC ± calcium. Western blot to glyceraldehyde-3-phosphate dehydrogenase (GAPDH) was used as the loading control. This section relates to Figure 7A. The membrane was cut in two pieces at the 50 kDa marker before blotting against GAPDH and the full membrane that was blotted is shown. Western blot was conducted once. **(B)** YTS cells were stained as in (A), except with 25 μM anti-CD45 NanoBondy. This section relates to Figure 7B. Western blot to GAPDH was used as the loading control. Western blot was conducted once.

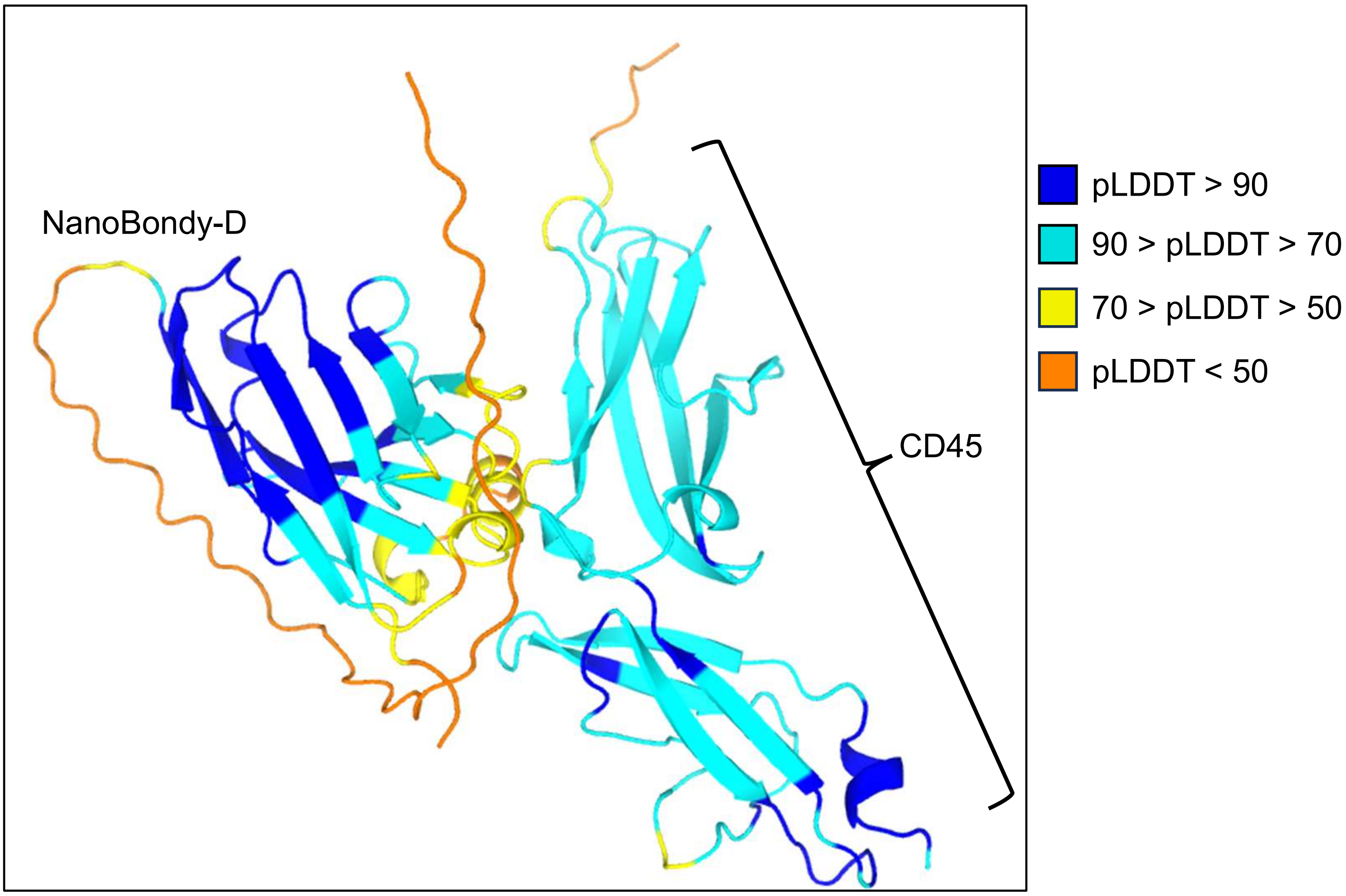

Supplementary Figure 7: AlphaFold2 prediction of NanoBondy/CD45d binding. AlphaFold2multimer prediction of 2H5 NanoBondy-D/CD45 structure, colored according to predicted Local Distance Difference Test (pLDDT) score. NanoBondy-D represents the NanoBondy following calcium-induced cleavage of the SPM.

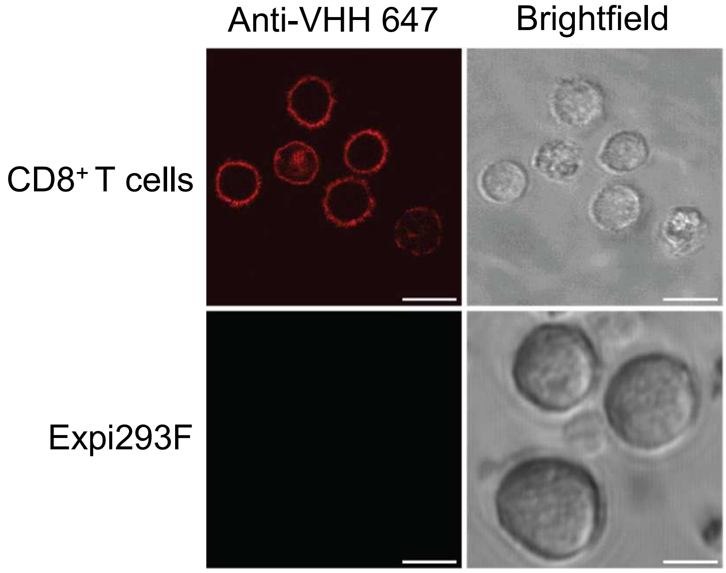

Supplementary Figure 8: Confocal microscopy of NanoBondy cell-surface labeling. Primary human CD8^{+} T cells (CD45^{+}) or Expi293F cells (CD45^{-}) were incubated with 2 µM anti-CD45 NanoBondy for 1 hour at 25 ºC. NanoBondy was detected using anti-VHH-Alexa Fluor 647 (shown in red, left column) with brightfield image shown alongside (grayscale, right column). Images shown are confocal slices. Scale bar is 10 µm. Microscopy experiment was conducted once.
